# Railway-Integrated Planning of Electric Vertical Takeoff and Landing Aircraft (eVTOL) Vertiports

**DOI:** 10.34133/research.1453

**Published:** 2026-10-23

**Authors:** Yitang Li, Hongxun Hui, Ling Zhan, Qimeng Zhang

**Affiliations:** ^1^ State Key Laboratory of Internet of Things for Smart City, University of Macau, Macao, China.; ^2^ Department of Electrical and Computer Engineering, University of Macau, Macao, China.; ^3^ Institute of Computing Technologies, China Academy of Railway Sciences Corporation Limited, Beijing, China.

## Abstract

Electric vertical takeoff and landing (eVTOL) aircraft provide a new and more sustainable form of mobility for passengers. In regional transport, eVTOL services can complement existing railway systems by providing direct air connections for selected trips. However, the practical value of eVTOL services is subject to strict infrastructure requirements beyond aircraft performance. Consequently, strategic vertiport deployment and charging capacity must be embedded into existing transport networks rather than planned as standalone systems. To achieve this objective, this paper presents a strategic railway-integrated planning framework for eVTOL vertiports and charging capacity. The framework treats railway stations as multimodal access nodes and leverages air links to selectively complement rail travel. It simultaneously optimizes siting, pad capacity, charging capacity, flight frequency, and fleet scale under infrastructure and operating constraints. To balance assignment fidelity and tractability in the integrated railway and air network, the framework applies a multicommodity flow (MCF) formulation for detailed network assignment and proposes a structured one-hop (SOH) formulation as a compact approximation. SOH restricts each substituted trip to a simple railway–air–rail (RAR) travel pattern with one direct air segment, thereby avoiding repeated intermodal transfers. Finally, the proposed framework is applied to scenario-based planning on a real-world railway network in Guangdong, China. The results show that all 11 sites selected by MCF are also selected by SOH, while SOH selects one additional site. The daily planning benefits obtained by MCF and SOH are 41,428 and 41,257 CNY/day, respectively, differing by only 0.4%, whereas SOH uses 93.5% fewer variables and reduces the solution time by 95.0%. Sensitivity analysis shows that no vertiport is selected when the transferable demand ratio is 1%. As the transferable demand ratio increases, both the planning benefit and the selected vertiport network increase until the site limit is reached.

## Introduction

Recent years have witnessed growing interest in the low-altitude economy and electric aviation, as cities and regions seek transport solutions that are faster, more flexible, and more compatible with long-term decarbonization and land use constraints [1,2]. In many densely populated corridors, conventional ground transport still faces structural limitations. Expanding rail or highway capacity often requires long construction cycles, high capital investment, and substantial right-of-way resources [3–5]. Even when such infrastructure is available, actual door-to-door travel efficiency remains limited by indirect routes, transfer frictions, and barriers in the terrain or built environment. These challenges are especially pronounced in metropolitan clusters and regional corridors, where strong travel demand coincides with the high cost and spatial constraints of constructing entirely new transport infrastructure.

Against this background, electric vertical takeoff and landing (eVTOL) aircraft provide a new and more sustainable form of mobility for passengers. This study focuses on their role in passenger transport. Advances in propulsion, flight control, aircraft configuration, and system-level energy design continue to improve eVTOL capability and endurance [6–8]. These aircraft offer vertical takeoff and landing capability, direct point-to-point operations, and reduced dependence on continuous linear infrastructure. With compact vertiport facilities, they can also be integrated with existing transport nodes to connect major activity centers more directly. For this reason, recent studies increasingly regard eVTOL as a complementary mode rather than a standalone replacement for conventional transport. Its practical value depends on how effectively it can be embedded into real multimodal networks and supported by feasible ground infrastructure, including pads, charging facilities, and fleet operations [9–12].

This perspective has shifted the focus of recent research from aircraft feasibility alone to the coordinated design of demand, infrastructure, operations, and multimodal integration [13–15]. The broader literature now recognizes that the introduction of urban air mobility (UAM) is a system problem involving transportation engineering, communication technology, energy infrastructure, and public acceptance. Recent reviews have shown that the field has evolved rapidly from discussions centered on technology toward integrated questions of demand, network design, infrastructure provision, operations, regulation, and sustainability [16,17]. Recent barrier analyses also emphasize that sustainable UAM deployment depends on coordinated attention to policy, operational, economic, and technical constraints [18]. At the same time, demand studies have suggested that potential adoption is highly heterogeneous across trip purposes and urban contexts. Previous work examined behavioral willingness to use UAM, estimated demand using machine learning and stated preference approaches, and highlighted that airport access, business travel, and selected regional trips may offer more realistic early markets than unrestricted citywide air taxi operations [19–23].

These findings shift attention from the aircraft alone to the supporting network and infrastructure. A central question therefore extends beyond technical feasibility to deployment locations, operating intensity, and supporting capacity requirements. Existing studies have investigated vertiport location, airport shuttle skyport planning, multimodal accessibility, equitable access, siting under obstacle constraints, integrated planning and operations, and multi-objective, multi-phased capacitated siting with land use integration [24–29]. Research on intercity low-altitude routes and public air route infrastructure further emphasizes coordination between low-altitude aviation, ground transport, and governance systems [16,30]. In parallel, UAM charging and scale-up studies show that repeated eVTOL operations depend on local electricity deliverability and charging coordination [31,32]; on the supply side, coordination of wind, solar, and storage under uncertainty provides the broader electricity system context for such demand [33]. Recent materials studies report advances in energy storage and environmental applications through composition and interface design [34–36]. Taken together, the planning and charging studies frame vertiport deployment as a coupled facility, capacity, energy, and fleet problem, while studies of the electricity supply and materials provide supporting technology context.

Despite this progress, an important planning setting remains relatively underexplored. Much of the current literature has treated UAM as a standalone urban service network or has focused on airport access and city taxi scenarios [22,37,38]. By contrast, many real intercity trips already rely on rail as the dominant mobility backbone. In such settings, the most meaningful role of eVTOL may be to complement railway travel by selectively replacing long, indirect, or time-intensive segments with direct air connections. This perspective is supported by the long-established literature on rail and air interaction, which has shown that multimodal coordination can reshape accessibility and corridor competitiveness when modes are combined strategically [39–41]. It also aligns with recent efforts to connect UAM planning with multimodal systems, including vertiport placement based on the topology of metropolitan rail networks and multimodal vertiport planning in large cities [42]. This study formalizes the deployment of eVTOL vertiports on an existing railway network while jointly sizing charging and operational support capacity.

This gap is important for both theory and practice. From a methodological perspective, railway-integrated eVTOL planning differs from conventional hub location and purely urban skyport design in several ways. The candidate access nodes already exist, their accessibility is determined by the railway network, and the value of an air connection depends on how much travel cost it can save relative to rail service [43–45]. Unlike standalone vertiport siting or siting based on land use, this setting makes the railway path and its detour structure part of every air link investment decision. It also differs from multimodal flow assignment in which infrastructure is fixed, because site activation, pad and charging capacity, flight frequency, and fleet scale are jointly optimized with passenger assignment. From a societal perspective, a railway-integrated design can leverage existing transport access points and target corridors where direct air links create meaningful time savings. These considerations define a strategic capacity planning problem for selectively upgrading railway stations as multimodal nodes [46–49].

The methodological contribution of this study is a railway-integrated planning formulation that couples passenger assignment with infrastructure and operating decisions in a mixed-integer linear programming (MILP) framework. Each candidate air link is evaluated against the existing railway path and the rail detour it can replace. The framework jointly optimizes origin–destination (OD) demand assignment, vertiport siting, pad and charging capacity, flight frequency, and fleet scale. We apply a multicommodity flow (MCF) formulation for detailed assignment and propose a structured one-hop (SOH) formulation for compact repeated analysis. MCF explicitly represents transferable OD demand in the integrated railway and air network, supporting detailed analysis of passenger flows, route choices, and multimodal interactions. SOH replaces free overlay network assignment with binary selection among beneficial railway–air–rail (RAR) schemes, each containing one direct air segment. This structure provides a compact approximation and a behaviorally interpretable representation of simple travel patterns that avoid repeated intermodal transfers, consistent with evidence that transfer counts materially affect passenger route choice [50].

The Results section first presents the planning framework and case study findings. The detailed mathematical formulations are provided in the Methods section.

## Results

### Problem overview of railway-integrated eVTOL vertiport planning

This study considers the planning of eVTOL vertiports on an existing railway network, where selected railway stations can be upgraded to support air services with associated charging capacity. The objective is to identify a cost-effective way to complement railway travel with air mobility while maintaining infrastructure feasibility and operational consistency [51,52].

Compared with conventional hub location or facility siting problems, the problem considered here exhibits 3 distinctive features. First, the air mobility system is not developed as a standalone network; instead, it is embedded into an existing railway system and serves as a complementary mode for selected OD demand pairs. Second, the planning decisions extend beyond binary siting choices to pad capacity, charging capacity, flight frequency, and fleet scale under station throughput limits. Third, the benefit of introducing eVTOL services depends on the interaction between railway accessibility, air travel time savings, and infrastructure feasibility, which jointly determine whether a candidate air connection is worthwhile. Consider a railway network represented by

$$G^{R}=\left(N,{\;A}^{R}\right)$$ (1)

where N is the set of railway stations and $A^{R}$ is the set of railway links. Each station i∈N is regarded as a candidate vertiport site for multimodal access. If selected, the station can provide eVTOL takeoff, landing, turnaround, and charging services.

The main planning decisions include the binary siting variable *x_i_*, which indicates whether station *i* is developed as a vertiport, the pad capacity variable *m_i_*, the charging capacity variable *s_i_*, the daily flight frequency *n_ab_*, and the fleet scale variable *F*. Together, these variables describe vertiport locations and the local service, charging capacity, and flight activity assigned to the selected multimodal network.

Given the set of selected vertiports, candidate air arcs can be established only between station pairs that satisfy the flight range and air route feasibility requirements. Let $A^{A}$ denote the set of feasible directed air arcs. Then, the integrated railway and air system can be represented by the overlay network

$$G=\left(N,{\;A}^{R}\cup A^{A}\right)$$ (2)

where railway links provide the ground access structure and air links provide potential bypass connections for selected travel demand.

Passenger demand is represented by a set of OD pairs P. For each OD pair p∈P, let $o_{p}$ and *d_p_* denote its origin and destination, respectively, and let *T_p_* denote its average daily total demand. In the integrated planning setting, the railway network remains the default mobility backbone, while eVTOL services are introduced as selective substitutes for part of the demand.

The overall structure of the proposed problem is illustrated in Fig. 1. As shown in the figure, the planning task starts from multimodal input data and engineering parameters, and is then formulated as a planning problem. To balance modeling fidelity and computational scalability, this study applies MCF and proposes SOH for the same planning problem. The framework is examined using a real-world railway network in Guangdong Province. As one of the most economically active and spatially diverse regions in China, it combines a dense metropolitan core with strong intercity demand potential and clear variation in railway corridor geometry. These features make it possible to examine where railway-integrated eVTOL services are most likely to generate value and how such value depends on local demand concentration and corridor structure.

**Fig. 1.**
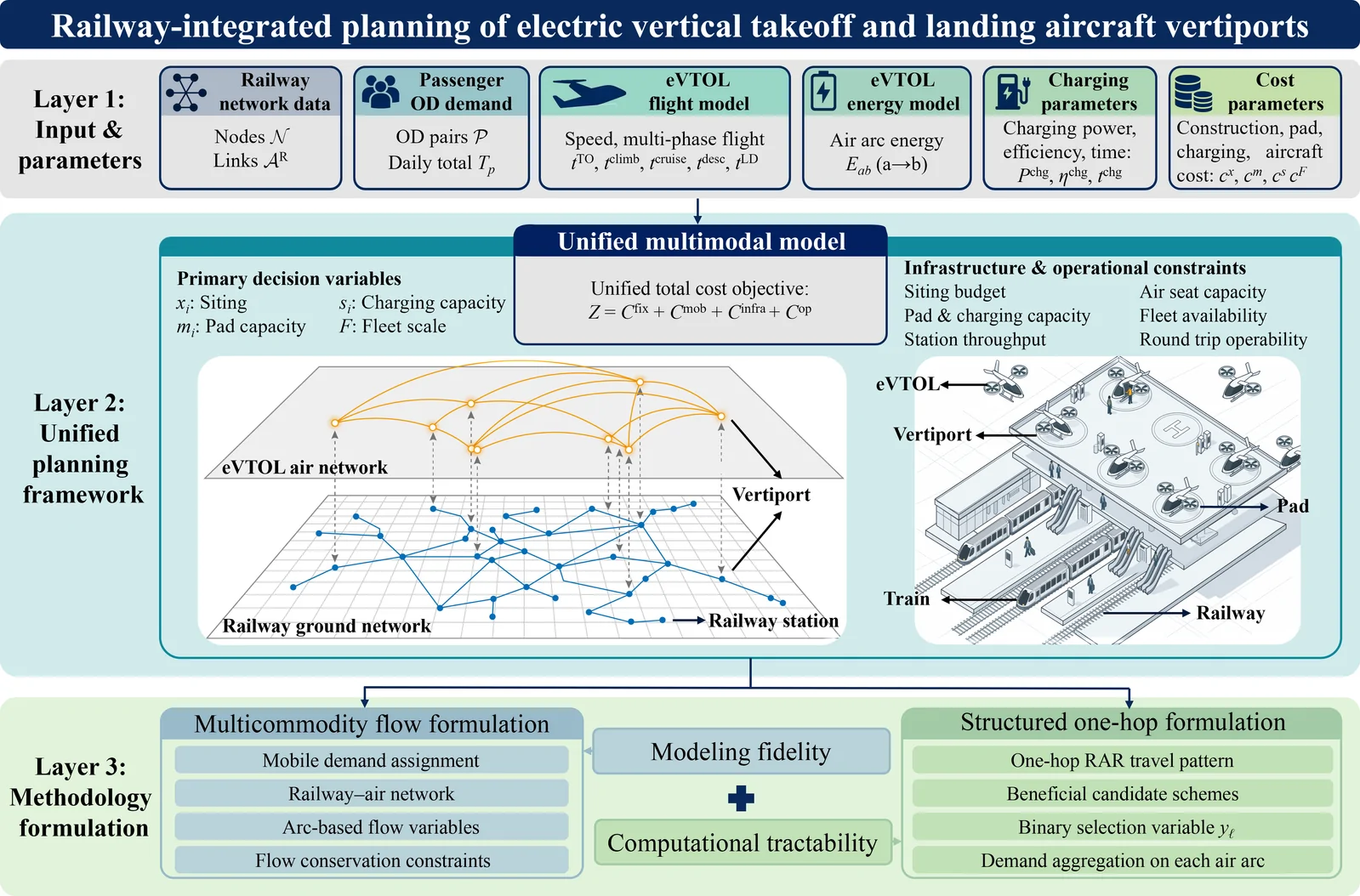
Framework of the strategic railway-integrated eVTOL planning problem. Inputs on the railway network, scenario demand, aircraft, cost, and feasibility are used in a unified model that jointly determines station activation, pad and charging capacity, flight frequency, and fleet scale through the MCF or SOH formulation.

### Case studies in Guangdong, China

The Guangdong case study provides a geographically grounded planning scenario for the framework introduced above. The regional railway network supplies the candidate node and link structure, while station-level population aggregation and a gravity model generate the scenario OD matrix. Its total demand is calibrated to regional railway statistics, and the resulting network supports scenario-based strategic analysis. Deployment validation would additionally require observed station-to-station passenger counts, land use and station function variables, and time-of-day trip records. The full station dataset, including station names, geographic coordinates, and station aggregated population, is provided in Table S1, while the railway link list used to construct the network topology is provided in Table S2.

Figure 2 illustrates the population density distribution of Guangdong Province together with the railway topology adopted in this study. The figure provides the geographical context of the planning problem and highlights the strong spatial heterogeneity of population concentration across the province. Highly urbanized corridors around the river delta exhibit dense population clusters and relatively dense railway connectivity, whereas peripheral regions show lower population density and more sparse rail coverage.

**Fig. 2.**
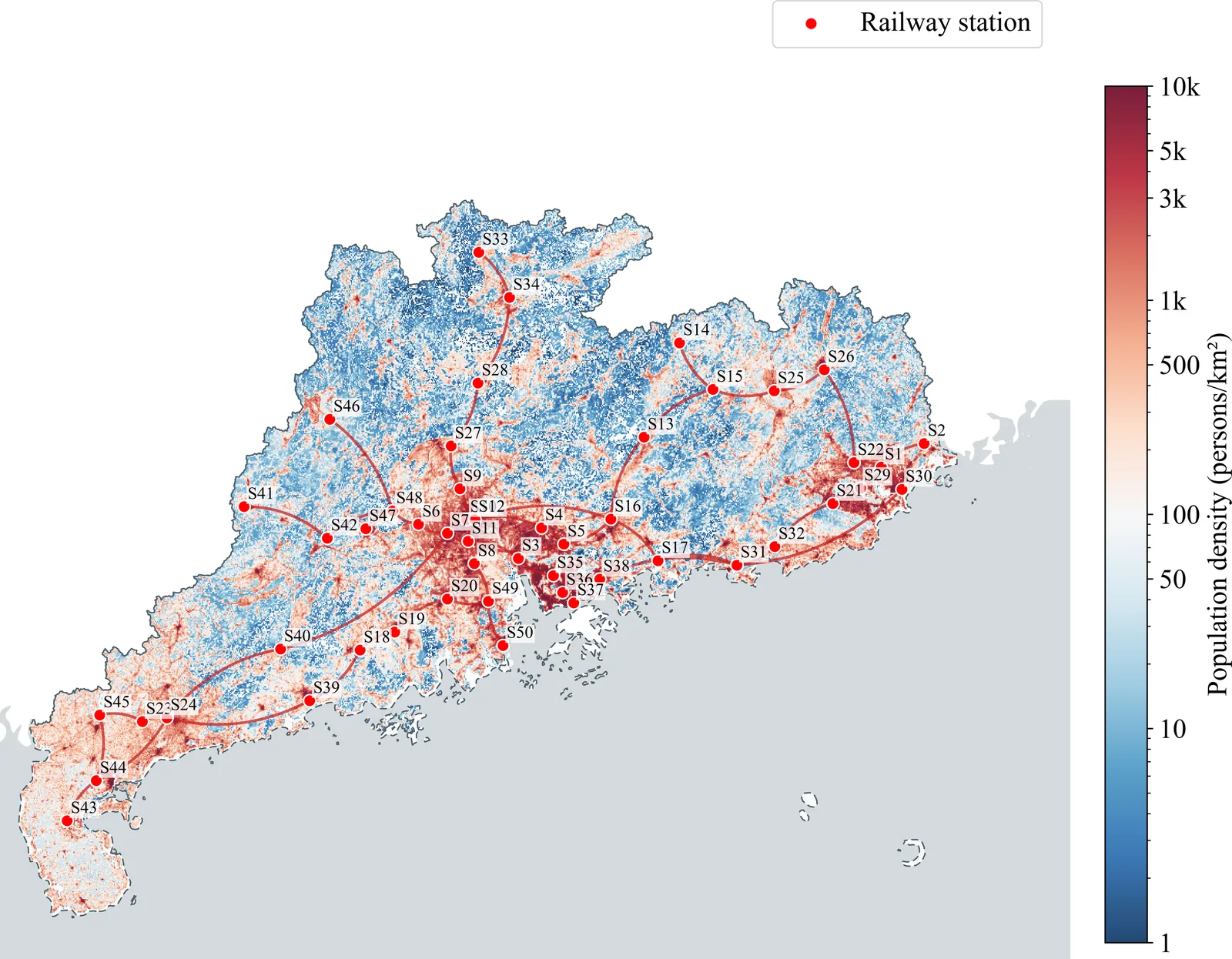
Population density distribution and railway topology in Guangdong Province. The heatmap shows the spatial distribution of population density, while the railway network and candidate stations provide the geographical basis for the subsequent eVTOL vertiport planning problem. Map approval no. GS(2019)3333.

To connect the continuous population field to the discrete railway planning model, the Voronoi partition of railway stations is constructed, and the population within each Voronoi cell is aggregated to its corresponding station. The resulting station population attraction is visualized in Fig. 3, where the bubble size represents the amount of population assigned to each railway node. The aggregated population assigned to each station is reported in Table S1. This step provides the node demand potential used in the subsequent OD demand construction and directly links the demographic distribution to the railway planning framework.

**Fig. 3.**
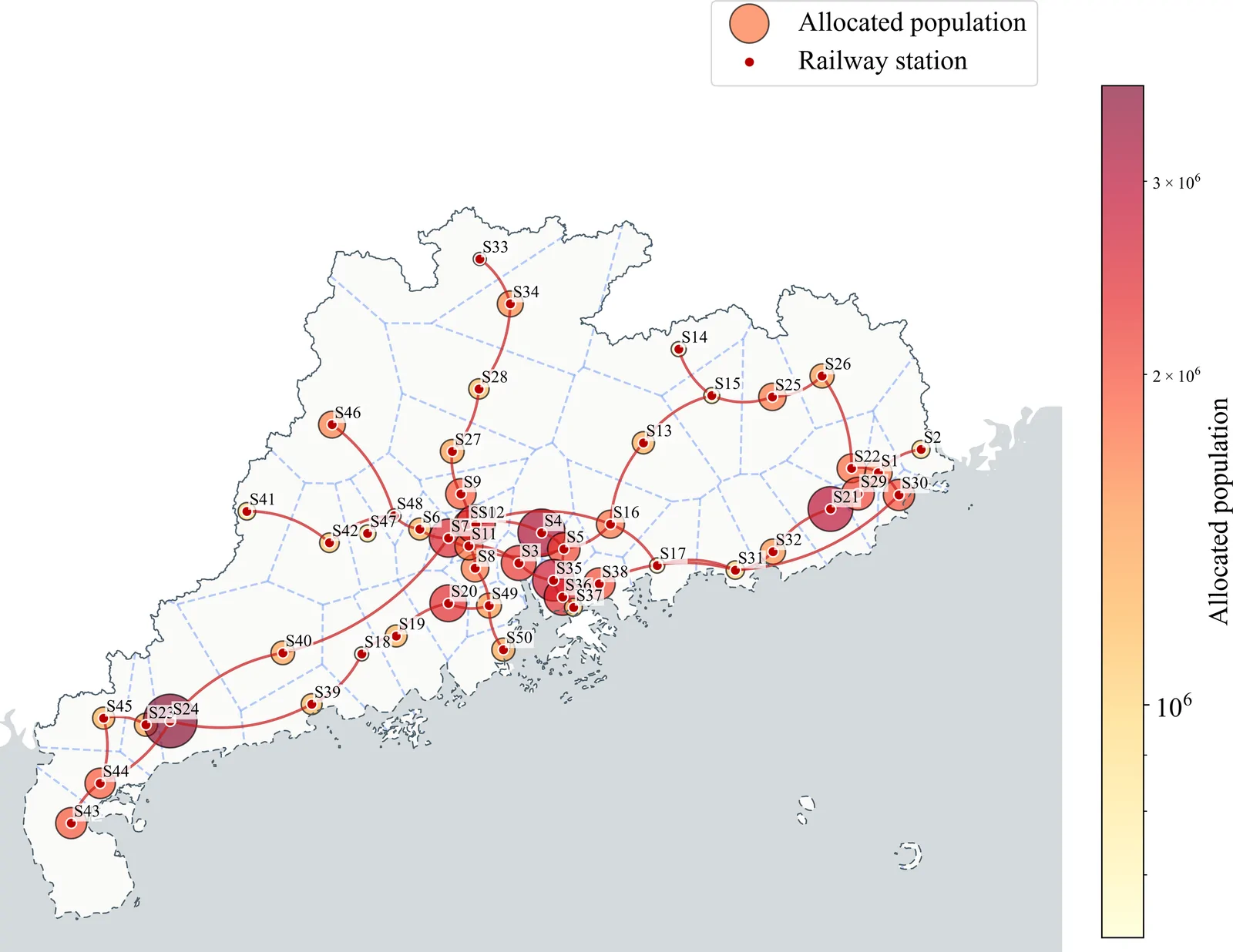
Population aggregation to railway stations using a Voronoi partition. Each Voronoi cell is associated with its nearest railway station, and the bubble size indicates the amount of population aggregated to that station. This station population representation is used to construct OD demand through the gravity model. Map approval no. GS(2019)3333.

Based on the station population aggregation, the average daily OD demand is generated through the gravity model formulation. The resulting OD matrix is visualized in Fig. 4 as a lower triangular heatmap, where symmetry is imposed by construction. The generated pattern concentrates demand among major urban centers and active intercity corridors. This concentration follows from the population, railway distance, and gravity model inputs. Within the planning scenario, it identifies where the optimization tests the substitution of rail travel with air service; observed OD flows would provide the corresponding deployment validation.

**Fig. 4.**
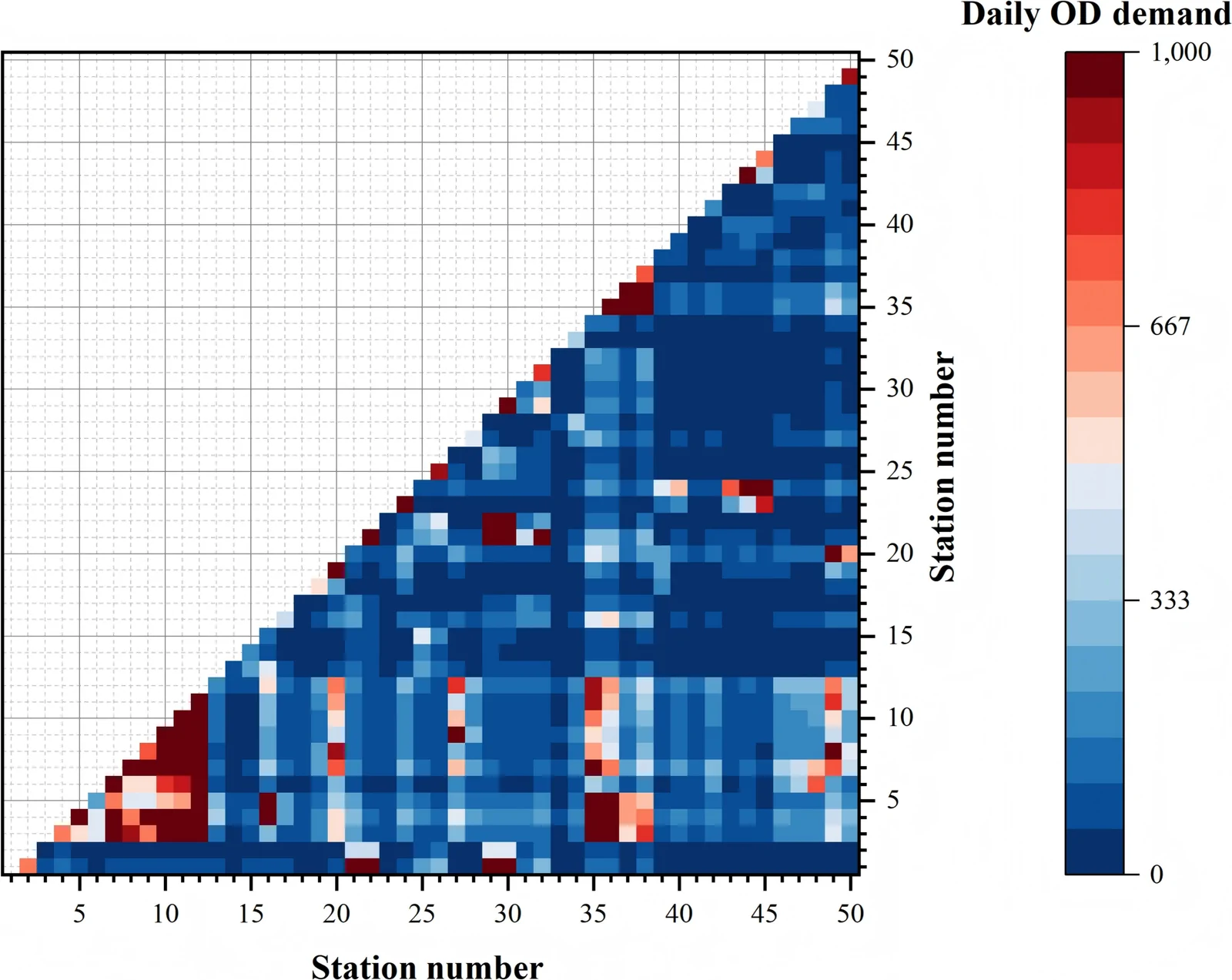
Average daily OD demand matrix for the planning scenario generated by the calibrated gravity model. The lower triangular heatmap is shown because the OD demand structure is symmetric. Larger values indicate stronger modeled demand between stations, and all values serve as planning inputs.

To further illustrate the structural motivation for introducing air mobility, Fig. 5 presents the lower triangular matrix of railway detour intensity, defined as the railway distance excess percentage over Euclidean distance. Values close to zero indicate that railway routing is nearly as direct as straight-line travel, whereas larger values indicate more substantial detours and thus stronger potential advantages for direct air connections. In this study, OD pairs with high ratios are of particular interest, since they represent locations where railway access remains available and direct air mobility may substantially reduce generalized travel cost.

**Fig. 5.**
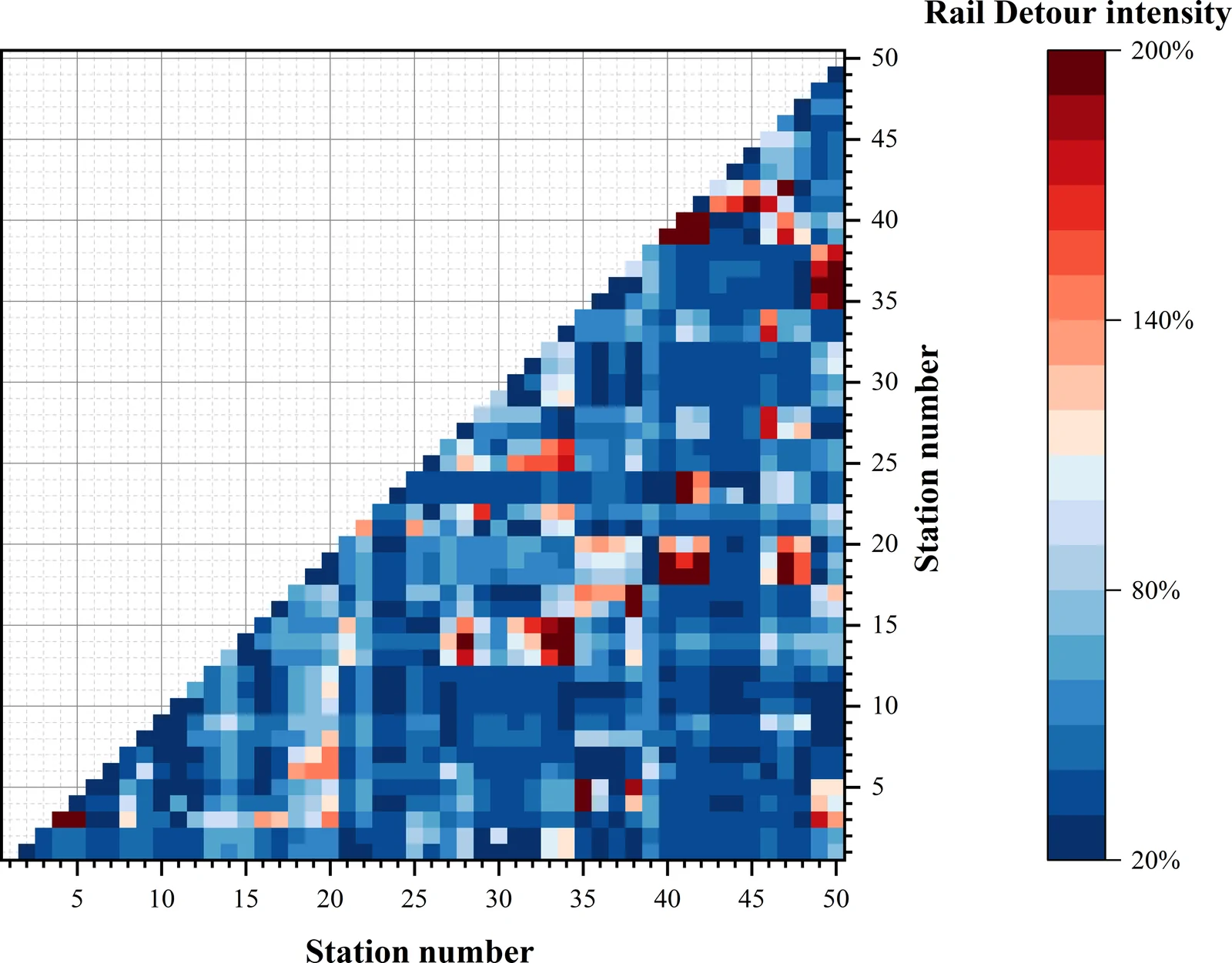
Lower triangular matrix of the railway distance excess percentage over Euclidean distance. Larger values indicate stronger rail detours relative to direct straight-line travel and thus greater potential convenience of direct air substitution.

Taken together, Figs. 4 and 5 explain the basic substitution logic of the planning problem: eVTOL services are most attractive when both OD demand intensity and rail detour intensity are sufficiently high. This also means that the subsequent optimization is not searching for vertiports uniformly across the whole railway network. Instead, it is expected to identify a limited number of high-value corridors in which strong travel demand and substantial rail detours appear at the same time. The optimization results presented later should therefore be interpreted as a selective corridor enhancement strategy rather than a provincewide coverage strategy.

### Planning results under the MCF and SOH planning scenarios

This subsection compares MCF and SOH planning scenarios using the same case settings. The MCF formulation represents detailed OD assignment and multimodal routing. The SOH formulation provides a compact RAR representation that avoids repeated transfers in scenario analysis. We compare their planning results and computational performance.

The deployment maps in Fig. 6A and B show the planning results at $K_{\max}=20$. MCF selects 11 vertiports. SOH selects the same sites and station 50. In both formulations, the selected vertiports and air routes are concentrated along major urban and intercity corridors in the Pearl River Delta. This pattern reflects the combined effects of travel demand and potential time savings relative to rail. The strong overlap shows that the main deployment pattern is consistent across the 2 formulations.

**Fig. 6.**
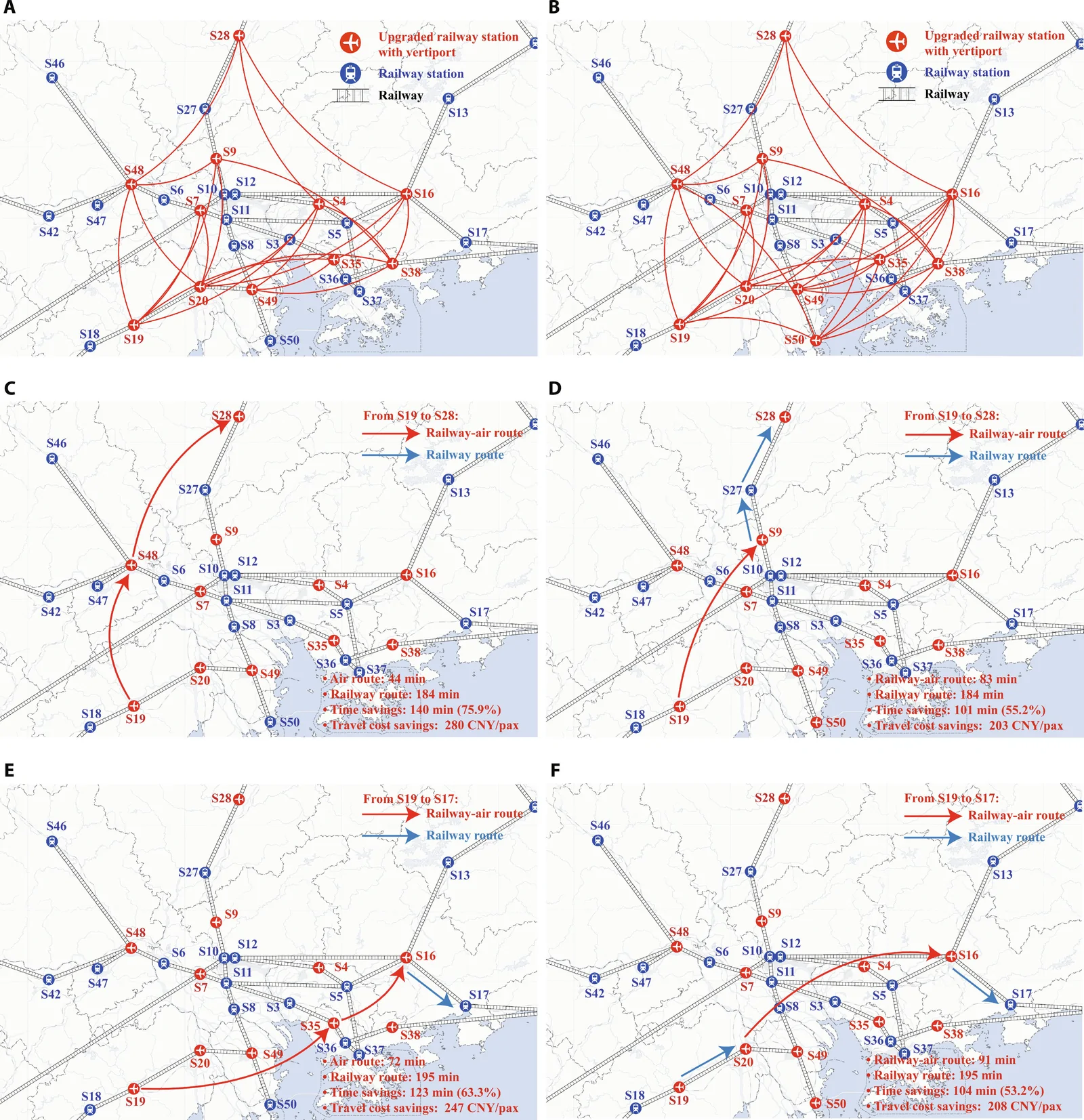
Optimized eVTOL vertiport siting, air route deployment, and comparisons of representative travel routes and times under the MCF and SOH planning scenarios. The railway network is shown in the background. Red arrows denote the optimized air or multimodal routes, and blue arrows denote the original railway routes. (A) Overall deployment under the MCF planning scenario. (B) Overall deployment under the SOH planning scenario. (C and D) Comparison of routes, travel times, and travel costs from station 19 to station 28 under the MCF and SOH planning scenarios, respectively. (E and F) Comparison of routes, travel times, and travel costs from station 19 to station 17 under the MCF and SOH planning scenarios, respectively. Map approval no. GS(2019)4342.

Figure 6C and D compares travel from station 19 to station 28. The original rail trip takes about 184 min. The shortest path in the MCF network takes about 44 min and uses 2 air segments, from station 19 to station 48 and from station 48 to station 28. This reduces travel time by 75.9% and saves about 280 CNY per passenger. The SOH path takes about 83 min and uses one air segment from station 19 to station 9, followed by rail travel through station 27 to station 28. This reduces travel time by 55.2% and saves about 203 CNY per passenger.

Figure 6E and F compares travel from station 19 to station 17. The original rail trip takes about 195 min. The MCF path takes about 72 min and uses air segments from station 19 to station 35 and from station 35 to station 16, followed by rail travel to station 17. The SOH path takes about 91 min and uses rail from station 19 to station 20, an air segment to station 16, and rail to station 17. The corresponding cost savings are about 247 and 208 CNY per passenger. Together, the deployment maps and route examples show that MCF and SOH produce similar spatial patterns with different levels of routing flexibility. MCF can use multiple air segments to identify more direct paths, while SOH still provides substantial benefits with one air segment.

Figure 7 compares travel times from station 1 to the other 49 stations under the original railway network and the networks produced by MCF and SOH at $K_{\max}=20$. Both optimized networks reduce travel time for 32 destinations. The savings are generally larger for medium and long trips, while many nearby destinations receive little or no benefit. The MCF network provides its largest savings for stations 18, 19, and 39, with a reduction of about 131 min. For example, travel time from station 1 to station 19 decreases from about 328 to 198 min. The largest SOH saving occurs at station 50, where travel time decreases from about 299 to 175 min. Stations 28, 33, and 34 also show savings of about 116 min. These results show that the benefits are concentrated on destinations with long or indirect rail routes rather than distributed evenly across the network.

**Fig. 7.**
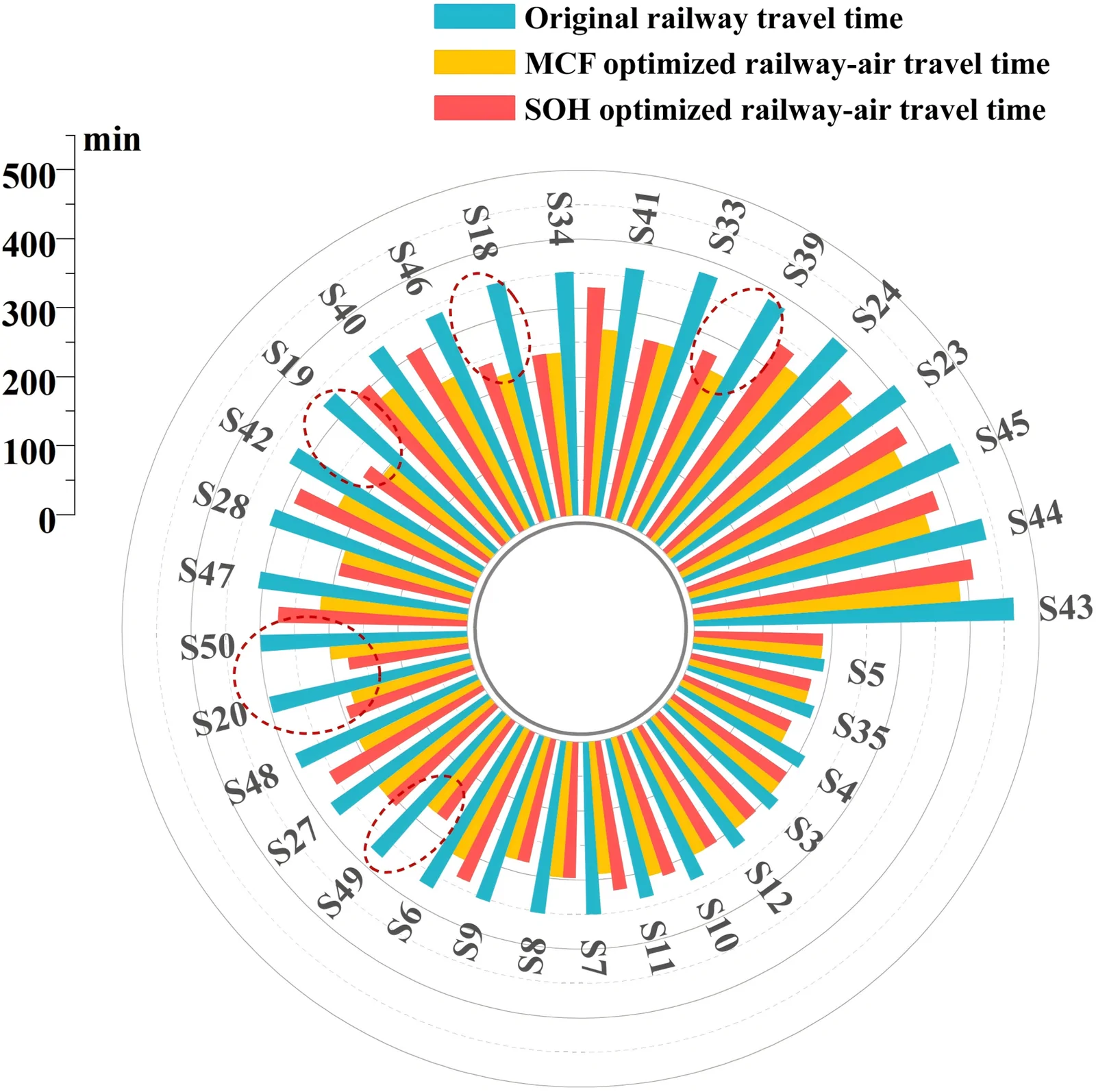
Travel times from station 1 to other stations under the original railway network and the optimized integrated networks generated by MCF and SOH. Destinations are ordered by their original railway travel times.

Figures 8 and 9 show the total OD daily flights between selected vertiports under MCF and SOH planning scenarios. The 5 strongest station pairs are the same in both results: Stations 38 and 49, stations 35 and 49, stations 16 and 35, stations 7 and 19, and stations 7 and 20. Under MCF, these pairs carry 59, 57, 50, 41, and 37 flights/day, respectively. Under SOH, the corresponding pairs are 61, 61, 49, 37, and 59 flights/day. Although their rankings and flights differ, both formulations identify the same main interaction corridors.

**Fig. 8.**
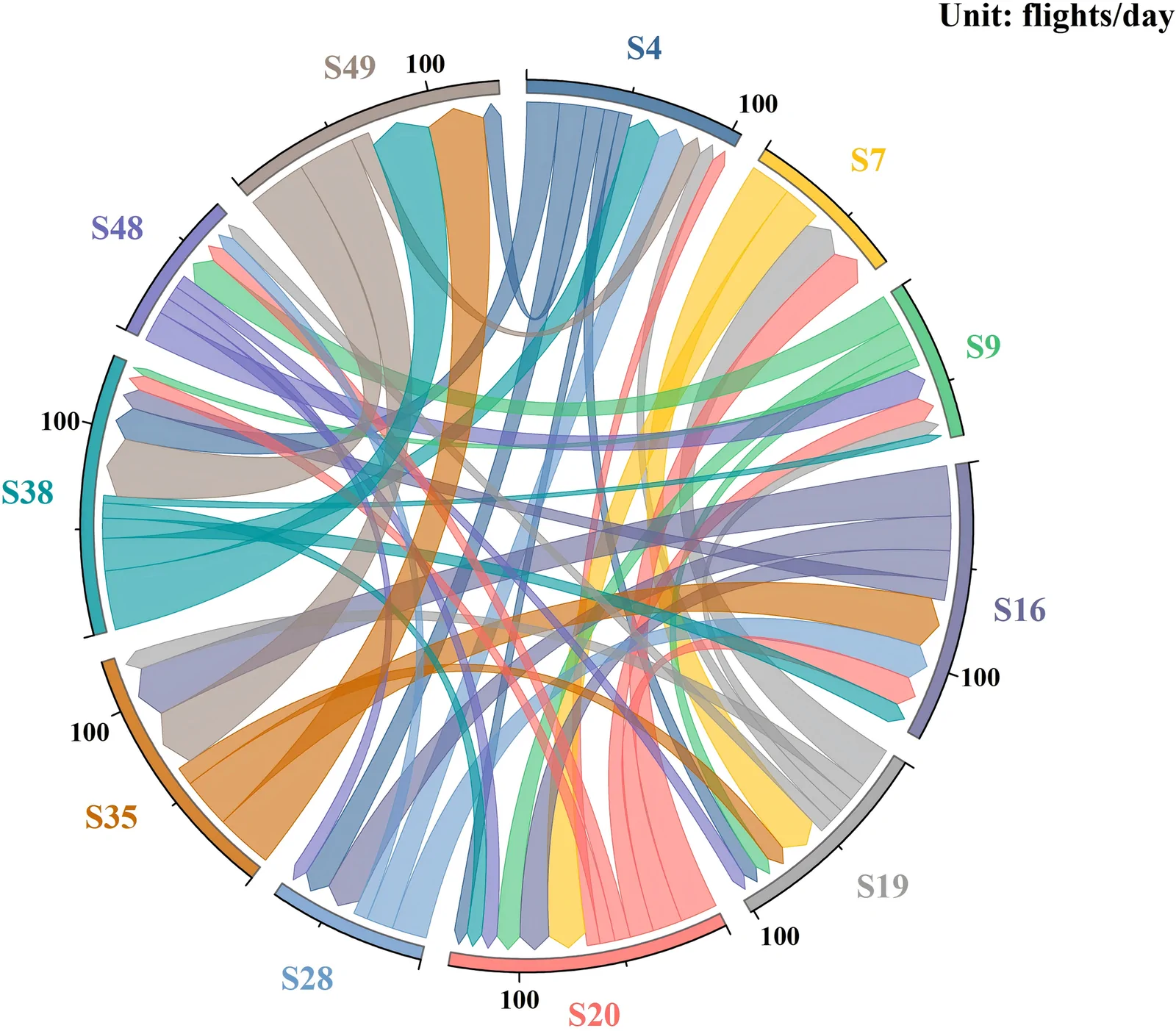
Chord diagram of OD daily flights among selected vertiports under the MCF planning scenario. Each node represents a selected vertiport, and each chord represents the total daily flights exchanged between a pair of vertiports. The strongest interaction is between stations 38 and 49, with 59 flights/day.

**Fig. 9.**
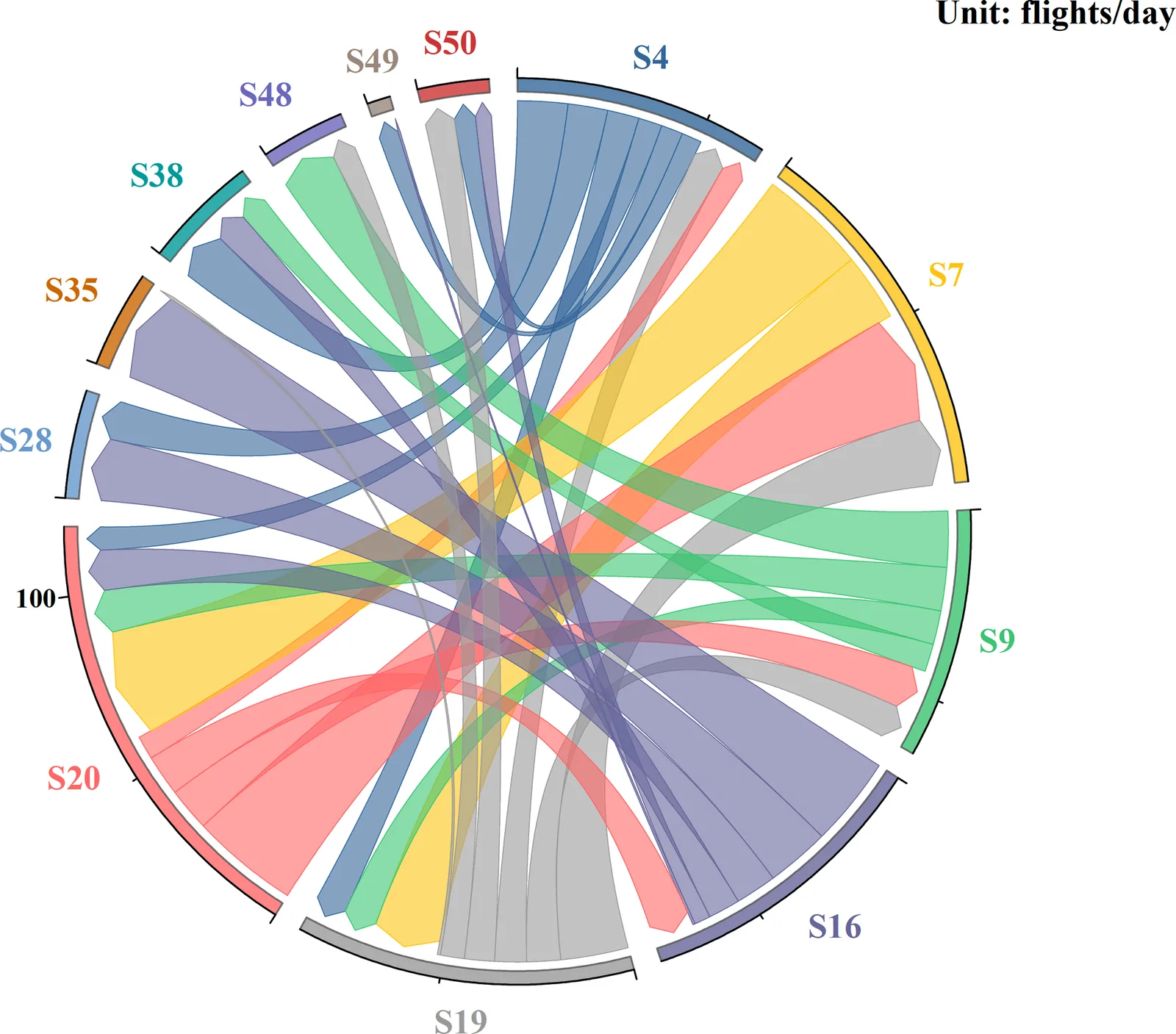
Chord diagram of OD daily flights among selected vertiports under the SOH planning scenario. Each node represents a selected vertiport, and each chord represents the total daily flights exchanged between a pair of vertiports. One of the strongest interactions is between stations 38 and 49, with 61 flights/day.

Table 1 reports the pad and charging capacities at the selected vertiports under MCF and SOH. Each site receives 2 to 4 pads, while charging capacity ranges from about 175 to 432 kW. Station 16 receives the largest charging capacity under both MCF and SOH, with 427 and 432 kW, respectively. Stations 19, 20, and 38 also receive more than 340 kW under both formulations. These results show that stations with similar pad capacities may require different charging capacities.

**Table 1.** Pad and charging capacities at the selected vertiports under MCF and SOH at *K*_max_ = 20

Station	MCF pads	MCF charging capacity (kW)	SOH pads	SOH charging capacity (kW)
Station 4	3	313	3	315
Station 7	2	175	3	201
Station 9	2	209	3	255
Station 16	4	427	4	432
Station 19	3	354	3	344
Station 20	4	396	4	343
Station 28	3	322	3	302
Station 35	3	278	3	256
Station 38	4	349	4	353
Station 48	2	189	2	206
Station 49	4	308	4	309
Station 50	–	–	2	216

Table 2 compares MCF and SOH at $K_{\max}=20$ under the same OD and cost settings. The selected sites are highly consistent. MCF selects 11 vertiports, while SOH selects 12, including all sites selected by MCF. SOH also assigns slightly greater total pad capacity, charging capacity, and fleet scale. Despite these differences, the 2 formulations produce similar overall results. MCF achieves a planning benefit of 41,428 CNY/day, only 0.4% higher than the 41,257 CNY/day obtained by SOH. Their total objective values are also close. The main difference is the solve time: MCF requires 907.9 s, while SOH requires 45.8 s, a reduction of 95.0%. These results support the use of SOH for repeated scenario analysis, while MCF remains useful for detailed OD assignment.

**Table 2.** Planning results and solve times for MCF and SOH at *K*_max_ = 20. Both formulations were solved on an Apple M3 computer with an 8-core CPU and 24 GB of memory using MATLAB R2026a and Gurobi 13.0.2. The relative mixed-integer programming (MIP) gap target was 10^−4^.

Metric	MCF	SOH
Selected sites	11	12
Total pad capacity	34	38
Total charging capacity (kW)	3,321	3,532
Fleet scale	55	59
Passengers served by air (pax/day)	2,336	2,551
Planning benefit (CNY/day)	41,428	41,257
Solve time (s)	907.9	45.8
Final relative MIP gap	0.0088%	0.0072%

### Sensitivity analysis and expanded network test

Sensitivity analyses are conducted for both MCF and SOH. The $K_{\max}$ analysis varies $K_{\max}\in \left\{5,10,12,15,20\right\}$ while fixing π = 0.05. The π analysis varies $\pi \in \left\{0.01,0.02,0.05,0.10,0.15,0.20\right\}$ while fixing $K_{\max}=20$. Both analyses use the same OD demand and cost settings as the main comparison. The $K_{\max}$ sensitivity results are reported in Table 3.

**Table 3.** Sensitivity to the vertiport site limit *K*_max_ at *π* = 0.05. Both formulations use the same OD matrix, aircraft parameters, and economic assumptions as the main comparison; planning benefits are reported in CNY/day.

*K*_max_	MCF benefit	SOH benefit	MCF sites	SOH sites
5	28,756	28,586	5	5
10	40,750	40,505	10	10
12	41,428	41,256	11	12
15	41,428	41,256	11	12
20	41,428	41,257	11	12

The planning benefit increases sharply as $K_{\max}$ rises from 5 to 10 and then changes little after $K_{\max}=12$. The increase from 5 to 10 sites is 11,994 CNY/day for MCF and 11,919 CNY/day for SOH, about 41.7% in both cases. Raising the limit from 10 to 12 adds only 678 CNY/day for MCF and 751 CNY/day for SOH. At $K_{\max}=12$, MCF selects 11 sites and achieves 41,428 CNY/day, while SOH selects 12 sites and achieves 41,256 CNY/day. Beyond $K_{\max}=12$, neither network expands and the planning benefit remains essentially unchanged. Raising the site limit no longer changes the solution because, under the baseline assumptions, the additional travel time savings from further sites are insufficient to offset their infrastructure and operating costs. The π sensitivity results are reported in Table 4.

**Table 4.** Sensitivity to the transferable demand ratio *π* at *K*_max_ = 20. Both formulations use the same OD matrix, aircraft parameters, and economic assumptions as the main comparison; planning benefits are reported in CNY/day.

*π*	MCF benefit	SOH benefit	MCF sites	SOH sites
0.01	0	0	0	0
0.02	1,731	1,799	2	2
0.05	41,428	41,257	11	12
0.10	141,462	144,220	20	19
0.15	203,202	211,646	20	20
0.20	255,017	264,082	20	20

At π = 0.01, neither formulation selects a vertiport and the planning benefit is zero. The transition between π = 0.02 and π = 0.05 is pronounced: The selected network grows from 2 sites to 11 sites under MCF and 12 sites under SOH as more OD pairs can support the fixed and capacity costs of air service. MCF reaches the limit of 20 sites at π = 0.10, while SOH reaches it at π = 0.15. Once both formulations have reached the 20-site limit, increasing π from 0.15 to 0.20 raises the planning benefit by 25.5% under MCF and 24.8% under SOH. The selected site count remains at 20, so these gains reflect more intensive use and reallocation within the selected network rather than continued spatial expansion. At π = 0.20, the planning benefits reach 255,017 CNY/day under MCF and 264,082 CNY/day under SOH. These results show that the transferable demand ratio affects both network formation and capacity use.

Additional paired sensitivity tests for individual factors evaluate both MCF and SOH under a common reference scenario. Operational range, usable battery capacity, rated charging power, and turnaround time are each evaluated at 5 levels. Both formulations show consistent directional patterns. Lower range or battery capacity can remove feasible air links. Higher charging power improves the planning benefit, while longer turnaround time reduces it. Among these 4 factors, charging power produces the largest change in planning benefit. Range and battery capacity have limited additional effects once useful air links become feasible. The complete paired results, including planning benefit, sites, fleet, site Jaccard index, and mixed-integer programming (MIP) gap for each formulation, are reported in Table S5.

Nine economic inputs are likewise evaluated at 5 levels in both MCF and SOH. Within the tested ranges, value of time produces the largest change in planning benefit. At its lowest tested value, neither formulation selects a vertiport; at the 2 highest values, both reach the site limit. Higher costs generally reduce planning benefit and can contract the selected network. Time and maintenance cost, grid energy cost, flight dispatch cost, and fleet investment cost have the largest effects among the cost factors. Both formulations agree on the endpoint direction for all 9 economic factors. Across the 45 paired cases, the median Jaccard index for the 2 selected site sets is 0.92, meaning that shared sites account for 92% of the union of the 2 sets in the median case. This high overlap indicates that MCF and SOH generally identify similar sets of sites, although some choices differ under extreme cost settings. The median absolute relative difference in planning benefit is 2.12%, measured against the MCF benefit. The complete paired economic results are reported in Table S6. These results show that network scale and site saturation depend on the economic scenario.

To examine computational performance beyond the original 50-node case, both formulations were tested on an 80-node expanded network. Both formulations use a relative MIP gap target of 0.1% under a 24-h time limit, with all other settings kept identical. The computational results are reported in Table 5.

**Table 5.** Computational results on the constructed 80-node expanded network at *K*_max_ = 20 and *π* = 0.05. Both formulations use a 0.1% relative MIP gap target and a 24-h time limit.

Formulation	Variables	Constraints	Status	Final gap	Solve time (s)
SOH	59,024	123,869	OPTIMAL	0.0980%	1,156.6
MCF	6,164,015	259,491	TIME LIMIT	2.4612%	86,400.5

SOH uses 99.04% fewer variables and 52.26% fewer constraints than MCF. It reaches the 0.1% target gap in 1,156.6 s. In contrast, MCF retains a gap of 2.46% after 24 h. The experiment demonstrates the computational effect of the compact one-hop representation for the tested network and parameter setting. MCF retains a larger routing space with higher fidelity, and larger applications may require decomposition, path generation, or other specialized strategies.

## Discussion

Railway-integrated eVTOL planning shifts attention from isolated vertiports and air links to the multimodal network. Within this network, eVTOL acts as a selective complement to rail rather than a replacement. A candidate station cannot be evaluated in isolation. Its value depends on its position in the railway network and the rail detour an air link can bypass. A corridor becomes viable only when demand and travel cost savings justify the required infrastructure and operating resources. The demand tests reveal 2 forms of network growth. Before the site limit binds, higher transferable demand supports more stations. After it binds, the selected network serves additional demand without adding sites. The proposed SOH represents simple travel patterns that avoid repeated transfers in a compact planning form. Its siting results closely align with MCF across the tested scenarios. This suggests that the main siting pattern can be retained without representing every path in equal detail. Together, these findings connect multimodal network design with capacity planning.

Deployment can proceed in stages and should be calibrated to local conditions. Early investment can focus on high-value station areas, while later expansion can follow observed demand. Aircraft performance and charging conditions should be evaluated for the local network before capital investment. Local value of time, fleet costs, and flight operating costs also require calibration. The Guangdong results provide a shortlist of candidate sites around railway stations for further assessment. This assessment should consider land, structural safety, obstacles, noise, emergency access, airspace, and regulatory approval. Deployment studies can replace the scenario OD matrix with observed demand data. The uniform transferable demand ratio can likewise be replaced by adoption estimates for individual OD pairs derived from stated preference or revealed preference data. These estimates can reflect passenger characteristics, service attributes, and local travel conditions. The framework focuses on strategic capacity planning rather than detailed operational scheduling. Future work can extend it to intraday operations with time windows and fleet routing. This extension can represent passenger waiting, charging queues, aircraft circulation, and repositioning across routes. Operational studies can then incorporate coordination with railway timetables, maintenance downtime, and stochastic delay. Larger networks will require scalable methods for detailed assignment.

## Methods

This section presents the methodological framework of the study. The objective is to support strategic planning of railway-integrated eVTOL vertiports together with local pad capacity, charging capacity, and fleet scale on an existing railway network. The model operates at a daily planning scale and uses aggregate capacity constraints to estimate infrastructure and resource requirements. It does not generate detailed timetables or aircraft schedules. The first formulation supports detailed flow analysis on the integrated railway and air network, while the second supports repeated strategic planning with improved computational efficiency.

### Study design of eVTOL vertiport planning

The study is designed to examine how eVTOL services can complement existing railway travel through selective air substitution. Guangdong Province is used as the case study region because it combines a dense metropolitan core, strong intercity demand, and substantial variation in railway corridor structure. The analysis proceeds in 4 steps. First, the railway network and regional population distribution are used to construct station demand and OD flows. Second, railway and air travel are represented through generalized travel cost, flight time, energy consumption, and charging time. Third, a planning problem is formulated to jointly optimize vertiport siting, pad capacity, charging capacity, and fleet scale under operational constraints and explicit site and route feasibility. Fourth, MCF is applied for detailed assignment, while the proposed SOH formulation provides a compact RAR representation that avoids repeated transfers in planning analysis.

### eVTOL flight and OD demand modeling

Let $d_{\mathrm{ab}}^{\mathrm{geo}}$ denote the geographic great circle distance between stations *a* and *b*. The range restriction applies to each eVTOL air segment rather than to the complete railway OD trip. The candidate air arc set is defined as

$$\begin{array}{c} A^{A}=\left\{\left(a,\;b\right):a,\;b\in N,\;a\neq b,{\;g}_{\mathrm{ab}}=1,{\;R}^{\min}\leq d_{\mathrm{ab}}^{\mathrm{geo}}\leq R^{\max}\right\} \end{array}$$ (3)

The candidate air arc set contains only station pairs that satisfy both the aircraft distance limits and the air route feasibility requirement. Here, $R^{\min}$ and $R^{\max}$ define the aircraft distance limits. The separate input $g_{\mathrm{ab}}$ determines whether an air route may be considered after assessment of airspace, safety, and regulatory requirements. Distance and range therefore do not establish air route feasibility on their own.

To reflect the operational characteristics of eVTOL flights, the air travel time is modeled through a multi-phase structure

$$t_{\mathrm{ab}}^{\mathrm{fly}}=t^{\text{TO}}+t^{\text{climb}}+t_{\mathrm{ab}}^{\text{cruise}}+t^{\text{desc}}+t^{\mathrm{LD}}$$ (4)

where $t^{\text{TO}}$, $t^{\text{climb}}$, $t^{\text{desc}}$, and $t^{\mathrm{LD}}$ are fixed phase durations, while $t_{\mathrm{ab}}^{\text{cruise}}$ is the cruise time determined by the distance of air arc $\left(a,\;b\right)$.The corresponding air arc energy consumption is modeled as

$$\begin{array}{c} \begin{array}{c} \begin{array}{c} E_{\mathrm{ab}}=P^{\text{TO}}t^{\text{TO}}+P^{\text{climb}}t^{\text{climb}}+P^{\text{cruise}}t_{\mathrm{ab}}^{\text{cruise}}+P^{\text{desc}}t^{\text{desc}}+P^{\mathrm{LD}}t^{\mathrm{LD}} \end{array} \end{array} \end{array}$$ (5)

where $P^{\text{TO}}$, $P^{\text{climb}}$, $P^{\text{cruise}}$, $P^{\text{desc}}$, and $P^{\mathrm{LD}}$ denote the power levels of the corresponding flight phases [53].

The charging time for arc $\left(a,\;b\right)$ is

$$t_{\mathrm{ab}}^{\mathrm{chg}}=\frac{E_{\mathrm{ab}}}{\eta^{\mathrm{chg}}P^{\mathrm{chg}}}$$ (6)

where $\eta^{\mathrm{chg}}$ is the charging efficiency and $P^{\mathrm{chg}}$ is the rated charging power [54]. These quantities provide the basis for the pad capacity, charging capacity, and fleet scale constraints used later in the planning model. Battery capacity further limits air arc feasibility through the condition $E_{\mathrm{ab}}\leq \left(1-\rho^{\mathrm{res}}\right)B^{\mathrm{use}}$, where $B^{\mathrm{use}}$ is usable battery capacity and $\rho^{\mathrm{res}}$ is the reserve fraction.

To construct the average daily OD demand on the railway network, this study first aggregates the population within each station catchment to the corresponding railway node. Let $w_{i}$ denote the aggregated population associated with station i∈N. For each ordered station pair $\left(i,\;j\right)$, the raw interaction intensity is generated by a gravity model form

$${\tilde{T}}_{\mathrm{ij}}=\frac{w_{i}w_{j}}{{\left(\max\left\{d_{\mathrm{ij}}^{R},1\right\}\right)}^{\alpha }},\;i\neq j$$ (7)

where $d_{\mathrm{ij}}^{R}$ denotes the railway shortest path distance between stations *i* and *j* in kilometers, and α is the distance decay parameter of the gravity model. The lower bound of 1 km is introduced only to avoid numerical instability for very short distances.

The raw interaction matrix is then normalized to match the total average daily railway demand $T^{\mathrm{tot}}$, yielding

$$T_{\mathrm{ij}}=T^{\mathrm{tot}}\frac{{\tilde{T}}_{\mathrm{ij}}}{\sum_{u\in N}\sum_{\begin{array}{c} v\in N\\ v\neq u \end{array}}{\tilde{T}}_{\mathrm{uv}}},\;i\neq j$$ (8)

Accordingly, for each OD pair p∈P with origin $o_{p}$ and destination $d_{p}$, the average daily total demand is defined as

$$T_{p}=T_{o_{p}d_{p}}$$ (9)

The calibration fixes the total demand scale, while the gravity model distributes it among station pairs. The resulting OD matrix supports analysis of planning scenarios.

Here, $p=\left(o_{p},{\;d}_{p}\right)$ denotes a directed OD pair. The reverse direction $p^{\prime }=\left(d_{p},{\;o}_{p}\right)$ is treated as a separate OD pair, even when the numerical demands in both directions are equal. In contrast, $\left(a,\;b\right)$ denotes a directed network arc from node *a* to node *b*. Thus, *p* indexes trip demand, while $\left(a,\;b\right)$ indexes movement on the network.

A key modeling assumption is that the same prescribed fraction of demand in every OD pair may consider eVTOL service. Let $\pi \in \left[0,\;1\right]$ denote this uniform transferable demand ratio. The total demand of OD pair *p* is decomposed into 2 components

$$T_{p}^{\mathrm{mob}}=\pi T_{p}$$ (10)

$$T_{p}^{\mathrm{fix}}=\left(1-\pi \right)T_{p}$$ (11)

Here, $T_{p}^{\mathrm{mob}}$ defines the upper bound on demand that may consider transfer from railway to air service, while $T_{p}^{\mathrm{fix}}$ is the fixed railway demand that remains on the railway network. The MCF assignment or SOH scheme selection determines realized air use within this upper bound according to generalized travel cost and the planning constraints.

Using the same ratio provides a common demand basis for comparing MCF and SOH under identical assumptions. The numerical values of the gravity model parameters and the eVTOL flight and charging parameters used in the case study are summarized in Table S3.

### Generalized travel cost and total cost formulation

Generalized travel cost is measured through travel time converted into economic cost. Ticket fares are not explicitly modeled because, from a social planning perspective, they mainly represent transfers between users and operators rather than net social costs. By contrast, travel time reflects a real welfare effect through passengers’ value of time, while infrastructure expenditures reflect real resource use. Accordingly, the generalized cost in this study is used to represent service quality and resource consumption rather than operator revenue.

For a railway arc $\left(a,\;b\right)\in A^{R}$, the generalized travel cost is defined as

$$c_{\mathrm{ab}}^{R}=\mathrm{VOT}\left(\frac{d_{\mathrm{ab}}^{R}}{v^{R}}+t^{\text{stop}}\right)$$ (12)

where $d_{\mathrm{ab}}^{R}$ is the effective railway distance, $v^{R}$ is the railway speed, $t^{\text{stop}}$ is the station stopping time, and VOT is the value of time. This parameter converts cumulative passenger travel time into monetary cost so that travel time, infrastructure cost, and operating cost can be evaluated in the same objective. In practical implementation, the railway distance is obtained by applying a circuity factor to the geographic straight-line distance so as to better approximate the real railway route.

For an air arc $\left(a,\;b\right)\in A^{A}$, the generalized travel cost is defined as

$$c_{\mathrm{ab}}^{A}=\mathrm{VOT}\;t_{\mathrm{ab}}^{\mathrm{fly}}$$ (13)

where $t_{\mathrm{ab}}^{\mathrm{fly}}$ denotes the eVTOL flight time.

Under the transferable demand approximation introduced above, the fixed railway demand contributes a constant railway cost term

$$C^{\mathrm{fix}}=\sum_{p\in P}T_{p}^{\mathrm{fix}}D_{o_{p}d_{p}}^{R}$$ (14)

where $D_{o_{p}d_{p}}^{R}$ denotes the shortest path generalized railway cost between the origin and destination of OD pair *p*.

The total system objective is then decomposed as

$$\min Z=C^{\mathrm{fix}}+C^{\mathrm{mob}}+C^{\text{infra}}+C^{\mathrm{op}}$$ (15)

where $C^{\mathrm{mob}}$ denotes the optimized generalized travel cost associated with the mobile demand component, $C^{\text{infra}}$ is the daily equivalent infrastructure investment cost, and $C^{\mathrm{op}}$ is the total operating cost of flights.

The infrastructure term is written as

$$\begin{array}{c} \begin{array}{c} C^{\text{infra}}=k^{\mathrm{day}}\left(\sum_{i\in N}c^{x}x_{i}+\sum_{i\in N}c^{m}m_{i}+\sum_{i\in N}c^{s}s_{i}+c^{F}F\right)+\sum_{i\in N}c^{\mathrm{om}}x_{i} \end{array} \end{array}$$ (16)

where $c^{x}$ is the fixed vertiport construction cost at station *i*; $c^{m}$ and $c^{s}$ are the unit costs of pad capacity and charging capacity, respectively; $c^{F}$ is the aircraft investment cost; $c^{\mathrm{om}}$ is the daily operation and maintenance cost of a selected vertiport; and $k^{\mathrm{day}}$ is the daily capital recovery factor derived from the discount rate and planning horizon.

Each selected vertiport incurs a common fixed base cost. Its total investment also includes the optimized pad and charging capacities through $c^{m}m_{i}$ and $c^{s}s_{i}$.

The mobile demand travel cost can be expressed abstractly as

$$C^{\mathrm{mob}}=\sum_{p\in P}T_{p}^{\mathrm{mob}}C_{p}\left(x\right)$$ (17)

where $C_{p}\left(x\right)$ denotes the minimum generalized travel cost experienced by the mobile demand of OD pair *p* under siting decision $x_{i}$. Since the feasible travel options depend on whether candidate air arcs are activated through vertiport construction, the cost term $C_{p}\left(x\right)$ is determined by the infrastructure design.

The operating cost of air services is further represented by a per flight cost term

$$C^{\mathrm{op}}=\sum_{\left(a,b\right)\in A^{A}}c_{\mathrm{ab}}^{\mathrm{op}}n_{\mathrm{ab}}$$ (18)

where $c_{\mathrm{ab}}^{\mathrm{op}}$ denotes the operating cost per flight on air arc $\left(a,\;b\right)$

$$c_{\mathrm{ab}}^{\mathrm{op}}=c^{\text{disp}}+\left(c^{\text{time}}+c^{\text{maint}}\right)t_{\mathrm{ab}}^{A}+c^{\mathrm{bat}}E_{\mathrm{ab}}+c^{\mathrm{ene}}\frac{E_{\mathrm{ab}}}{\eta^{\mathrm{chg}}}+c^{\mathrm{km}}d_{\mathrm{ab}}^{A}$$ (19)

where $c^{\text{disp}}$ is a fixed dispatch and ground service cost per flight, $c^{\text{time}}$ and $c^{\text{maint}}$ are operating and maintenance cost coefficients based on time, $c^{\mathrm{bat}}$ and $c^{\mathrm{ene}}$ are battery and energy cost coefficients, $\eta^{\mathrm{chg}}$ is the charging efficiency, and $c^{\mathrm{km}}$ is an optional operating cost coefficient based on distance. Here, $t_{\mathrm{ab}}^{A}=t_{\mathrm{ab}}^{\mathrm{fly}}$ and $d_{\mathrm{ab}}^{A}=d_{\mathrm{ab}}^{\mathrm{geo}}$ denote flight time and air segment distance, respectively.

In the numerical results, this study reports a planning benefit indicator, defined as the reduction in total system cost relative to the all-rail baseline

$$B=Z^{\text{rail}}-Z$$ (20)

where $Z^{\text{rail}}$ denotes the total system cost under the all-rail baseline without eVTOL deployment, and *Z* is the optimized total cost under the integrated planning model.

### Infrastructure and operational constraints

Although the 2 formulations differ in how the mobile demand is assigned, they share the same infrastructure and operational feasibility structure.

First, the number of selected vertiports is limited by the upper bound $K_{\max}$ imposed by the siting budget

$$\sum_{i\in N}x_{i}\leq K_{\max}$$ (21)

Second, site feasibility is enforced by

$$x_{i}\leq h_{i},\;\forall i\in N$$ (22)

Here, $h_{i}$ indicates whether a station area is available for consideration in the planning model. The selected locations represent candidate sites around railway stations. Before implementation, their local feasibility should be determined from land and structural conditions, obstacle and noise constraints, emergency access, and regulatory approval.

Third, pad capacity and charging capacity can only be installed at selected vertiports

$$m_{i}\leq {\bar{m}}_{i}x_{i},\;\forall i\in N$$ (23)

$$s_{i}\leq {\bar{s}}_{i}x_{i},\;\forall i\in N$$ (24)

where ${\bar{m}}_{i}$ and ${\bar{s}}_{i}$ denote the upper bounds of pad capacity and charging capacity at station *i*. For every feasible air arc $\left(a,\;b\right)\in A^{A}$, the passenger demand assigned to that arc must not exceed its seat capacity

$$q_{\mathrm{ab}}^{A}\leq Q\;n_{\mathrm{ab}},\;\forall \left(a,\;b\right)\in A^{A}$$ (25)

where $q_{\mathrm{ab}}^{A}$ denotes the passenger volume assigned to air arc $\left(a,\;b\right)$, and *Q* is the number of seats per flight.

The relationship between transferable demand, air arc passenger volume, and flight frequency is explicit. $T_{p}^{\mathrm{mob}}$ defines the potentially transferable demand of directed OD pair *p*. The MCF or SOH assignment aggregates the portion using air service into $q_{\mathrm{ab}}^{A}$, the passenger volume on directed air arc $\left(a,\;b\right)$. Equation (25) then determines the minimum flight frequency required to provide sufficient seats for this passenger volume.

Fourth, the total pad occupancy induced by departures, arrivals, turnaround, and charging must remain within the daily service capability

$$\begin{array}{c} K^{\text{peak}}\left[\sum_{\left(a,b\right)\in A^{A}:a=i}n_{\mathrm{ab}}t^{\text{prep}}+\sum_{\left(a,b\right)\in A^{A}:b=i}n_{\mathrm{ab}}\left(t^{\text{turn}}+t_{\mathrm{ab}}^{\mathrm{chg}}\right)\right]\\ \leq \rho^{m}{\mathrm{Hm}}_{i},\;\forall i\in N \end{array}$$ (26)

where $K^{\text{peak}}$ is the peak demand amplification factor, $t^{\text{prep}}$ is the pre-departure pad occupation time, $t^{\text{turn}}$ is the turnaround time after arrival, $\rho^{m}$ is the allowable pad utilization factor, and *H* is the effective daily operating time. The factor converts daily flight activity into peak equivalent capacity demand. It is applied to pad, charging, fleet, and station throughput constraints. $K^{\text{peak}}$ reflects the ratio of peak activity to average activity, allowing infrastructure and fleet resources to be sized for concentrated peak operations.

Fifth, charging capacity must be sufficient to support the total energy demand after arrival

$$K^{\text{peak}}\sum_{\left(a,b\right)\in A^{A}:b=i}n_{\mathrm{ab}}E_{\mathrm{ab}}\leq {\mathrm{Hs}}_{i},\;\forall i\in N$$ (27)

Sixth, the total aircraft cycle time must be supportable by the available fleet scale

$$K^{\text{peak}}\sum_{\left(a,b\right)\in A^{A}}n_{\mathrm{ab}}\left(t_{\mathrm{ab}}^{\mathrm{fly}}+t_{\mathrm{ab}}^{\mathrm{chg}}+t^{\text{prep}}+t^{\text{turn}}\right)\leq \rho^{F}\mathrm{HF}$$ (28)

where $\rho^{F}$ is the allowable fleet utilization factor and *H* is the daily operating duration.

Seventh, each selected vertiport is subject to an hourly station throughput limit. Let $\bar{\mu }$ denote the maximum total number of arrival and departure movements that a station can handle per hour. The hourly throughput constraint is written as

$$K^{\text{peak}}\left[\sum_{\left(a,b\right)\in A^{A}:a=i}n_{\mathrm{ab}}+\sum_{\left(a,b\right)\in A^{A}:b=i}n_{\mathrm{ab}}\right]\leq \bar{\mu }x_{i}H,\;\forall i\in N$$ (29)

This constraint limits the peak hourly aircraft movements that can be handled by each selected vertiport.

Finally, air services in opposite directions are balanced to maintain approximate round trip operability

$$n_{\mathrm{ab}}=n_{\mathrm{ba}},\;\forall \left(a,\;b\right)\in A^{A}$$ (30)

This equality is a planning approximation for round trip operability. It does not model time-dependent fleet circulation or aircraft repositioning across routes.

Together, Eqs. (21) to (30) define the feasible region for vertiport siting, pad capacity, charging capacity, station throughput, flight frequency, and fleet scale. The related parameter settings of the optimization are summarized in Table S4.

The planning problem is formulated in 2 ways to support different planning scenarios. As summarized in Fig. 10, both formulations are built on the same demand splitting assumption, the same feasibility constraints, and the same total cost objective. They differ in how the mobile demand component is represented. The MCF formulation supports detailed planning on the integrated railway and air network. The proposed SOH formulation supports repeated planning analysis through compact selection of RAR schemes that avoid repeated transfers. The MCF formulation is introduced first, followed by the SOH formulation.

**Fig. 10.**
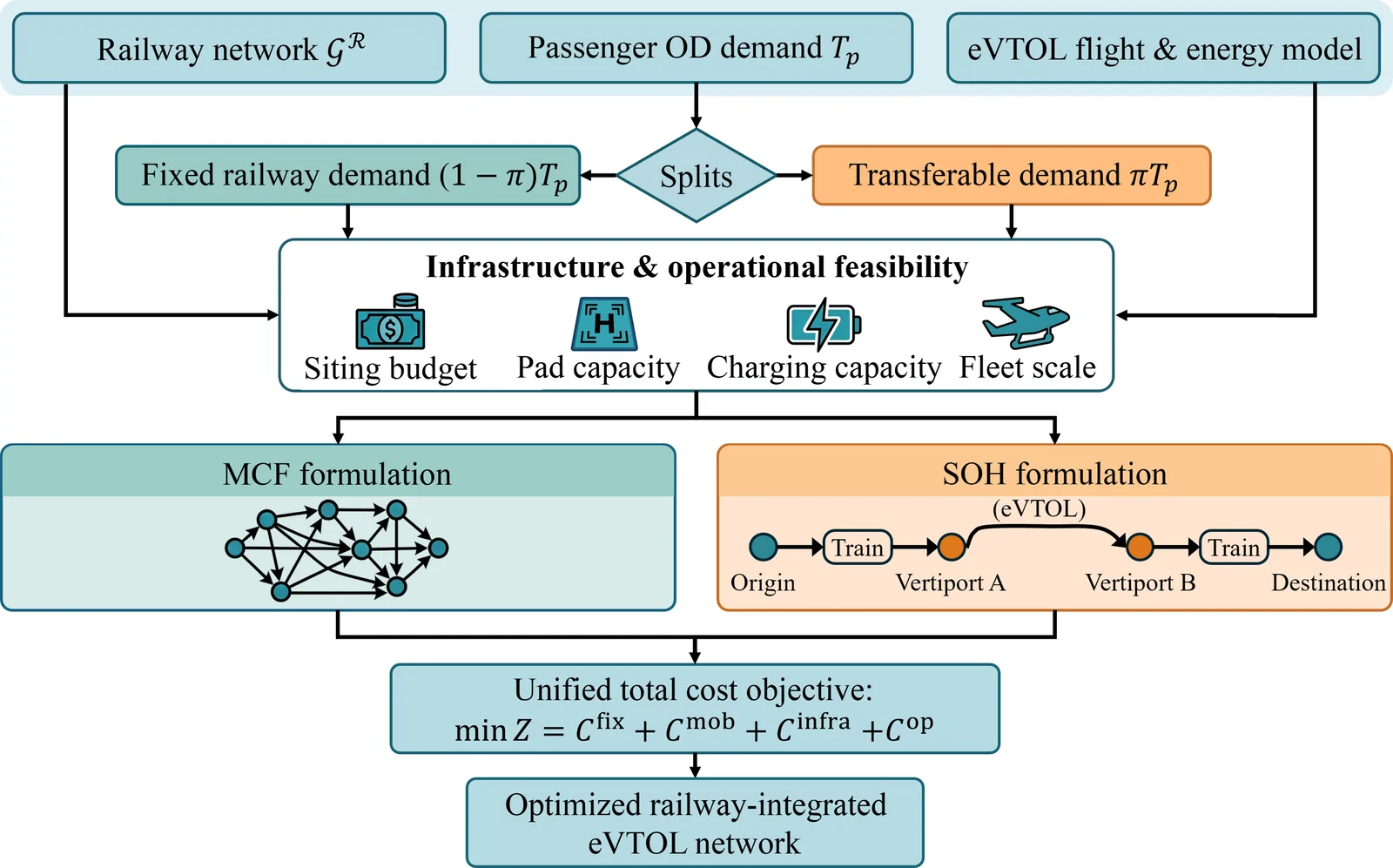
Relationship between the railway-integrated eVTOL planning problem and its 2 MILP formulations. Both formulations are derived under the same demand splitting assumption, infrastructure and operational constraints, and total cost objective.

### MCF formulation for detailed planning

Under the planning framework introduced in the Problem overview of railway-integrated eVTOL vertiport planning section, only the mobile demand component $T_{p}^{\mathrm{mob}}$ is subject to mode reallocation, while the fixed railway demand component $T_{p}^{\mathrm{fix}}$ is treated as a constant railway baseline term in the total objective. The MCF formulation assigns mobile demand on the integrated railway and air network, as shown in Fig. 11. Therefore, the route choice component determined by the model in Eq. (17) is converted into explicit flow variables for each arc and linear flow conservation constraints.

**Fig. 11.**
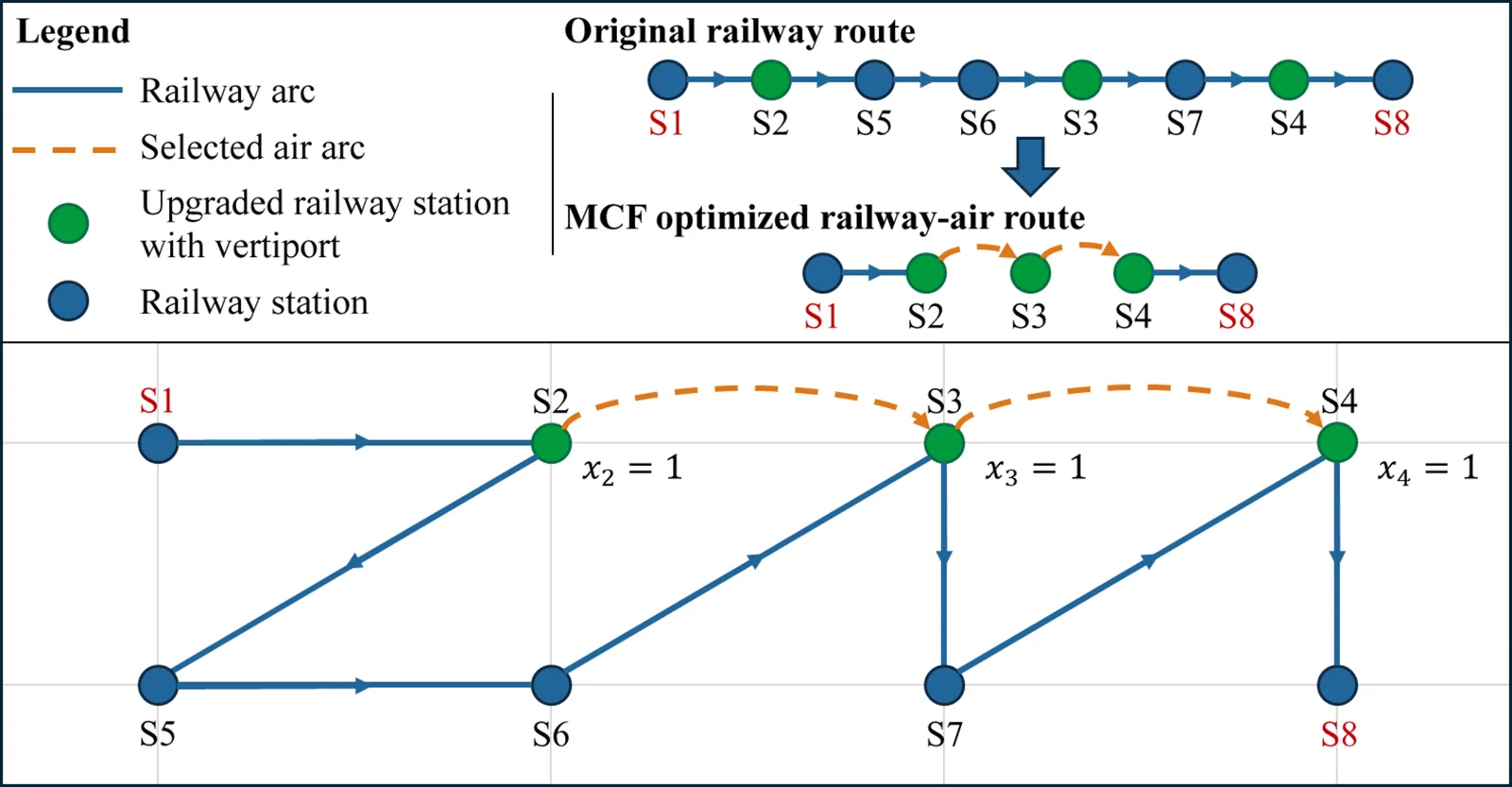
Illustration of mobile demand assignment under the MCF formulation on the integrated railway and air network. The upper panel compares the original railway route with the MCF optimized route. The lower panel shows the overlay network consisting of railway arcs and air arcs, where selected stations are upgraded to vertiports and mobile demand is assigned through MCFs.

For each OD pair p∈P and each arc $e\in A^{R}\cup A^{A}$, define the nonnegative flow variable

$$f_{e,p}\geq 0,\;\forall e\in A,\;\forall p\in P$$ (31)

which represents the portion of mobile demand $T_{p}^{\mathrm{mob}}$ assigned to arc *e*.

An air service can only be operated if both endpoint stations are selected as vertiports

$$n_{\mathrm{ab}}\leq {\mathrm{Mx}}_{a},\;\forall \left(a,\;b\right)\in A^{A}$$ (32)

$$n_{\mathrm{ab}}\leq {\mathrm{Mx}}_{b},\;\forall \left(a,\;b\right)\in A^{A}$$ (33)

where *M* is a sufficiently large constant.

The assignment of mobile demand is governed by standard MCF conservation constraints. For each node i∈N and OD pair p∈P,

$$\begin{array}{c} \begin{array}{c} \sum_{e\in \delta^{+}\left(i\right)}f_{e,p}-\sum_{e\in \delta^{-}\left(i\right)}f_{e,p}=\left\{\begin{array}{cc} T_{p}^{\mathrm{mob}}, & i=o_{p},\\ -T_{p}^{\mathrm{mob}}, & i=d_{p},\\ 0, & \text{otherwise}, \end{array}\right.\;\forall i\in N,\;\forall p\in P \end{array} \end{array}$$ (34)

where $\delta^{+}\left(i\right)$ and $\delta^{-}\left(i\right)$ denote the sets of outgoing and incoming arcs of node *i*, respectively.

The MCF flow conservation constraint assigns the potentially transferable demand between its origin and destination on the integrated railway and air network. The demand can use an all-rail path or a path containing air arcs; only flow assigned to air arcs represents realized air use.

The aggregate passenger volume assigned to each air arc is linked to the OD MCF variables by

$$q_{\mathrm{ab}}^{A}=\sum_{p\in P}f_{\left(a,b\right),p},\;\forall \left(a,\;b\right)\in A^{A}$$ (35)

Together with the shared capacity [Eq. (25)], this ensures that MCF air arc flow and flight frequency are expressed in passengers per day and flights per day, respectively.

Since the fixed railway demand term $C^{\mathrm{fix}}$ has already been separated in the Generalized travel cost and total cost formulation section, the MCF formulation only needs to optimize the travel cost associated with the mobile demand component. Under the flow representation by arc, this term becomes

$$C_{\left(1\right)}^{\mathrm{mob}}=\sum_{p\in P}\sum_{e\in A}c_{e}f_{e,p}$$ (36)

where $c_{e}$ denotes the generalized cost of arc *e*, as defined in the Generalized travel cost and total cost formulation section.

Accordingly, the MCF formulation minimizes

$$Z^{\left(1\right)}=C^{\mathrm{fix}}+C_{\left(1\right)}^{\mathrm{mob}}+C^{\text{infra}}+C^{\mathrm{op}}$$ (37)

Since $C^{\mathrm{fix}}$ is constant for a given demand set, the optimizer acts on the remaining cost components. The final total cost retains $C^{\mathrm{fix}}$ for consistency with the objective in the Generalized travel cost and total cost formulation section.

The MCF formulation is obtained by combining the mobile demand flow [Eqs. (31) to (35)] with the infrastructure and operational feasibility [Eqs. (21) to (30)] in the previous section. The resulting formulation is a MILP in which assignment for each OD pair remains explicitly represented through MCFs.

The main advantage of the MCF formulation is its relatively high modeling fidelity. Since the transferable demand is assigned directly over the integrated railway and air network, the formulation can capture a broad set of multimodal path combinations without predefining route templates. At the same time, this fidelity comes at the cost of model size, because the number of continuous flow variables scales with ∣A∣∣P∣. As a result, the MCF formulation is best suited to detailed planning settings in which OD interaction patterns need to be interpreted explicitly. This also motivates the need for a more compact formulation for repeated strategic planning, which is introduced next.

### SOH formulation for compact scenario analysis

The SOH formulation is designed for compact strategic planning, where the main objective is to identify major substitution corridors and priority vertiport locations with lower computational cost. Unlike the MCF formulation, which allows mobile demand to move on the full integrated railway and air network, the SOH formulation adopts a more structured representation of substituting rail travel with air service. This design reduces model size and makes repeated scenario analysis more practical on larger networks.

Specifically, the SOH formulation assumes that the mobile demand component $T_{p}^{\mathrm{mob}}$ may use air mobility through a single direct air segment embedded in a RAR travel pattern. Under this setting, free path assignment across the network is replaced by binary candidate scheme selection, as illustrated in Fig. 12. The resulting model is more compact than the MCF formulation while preserving the main planning logic of selective air substitution between strategically chosen railway stations.

**Fig. 12.**
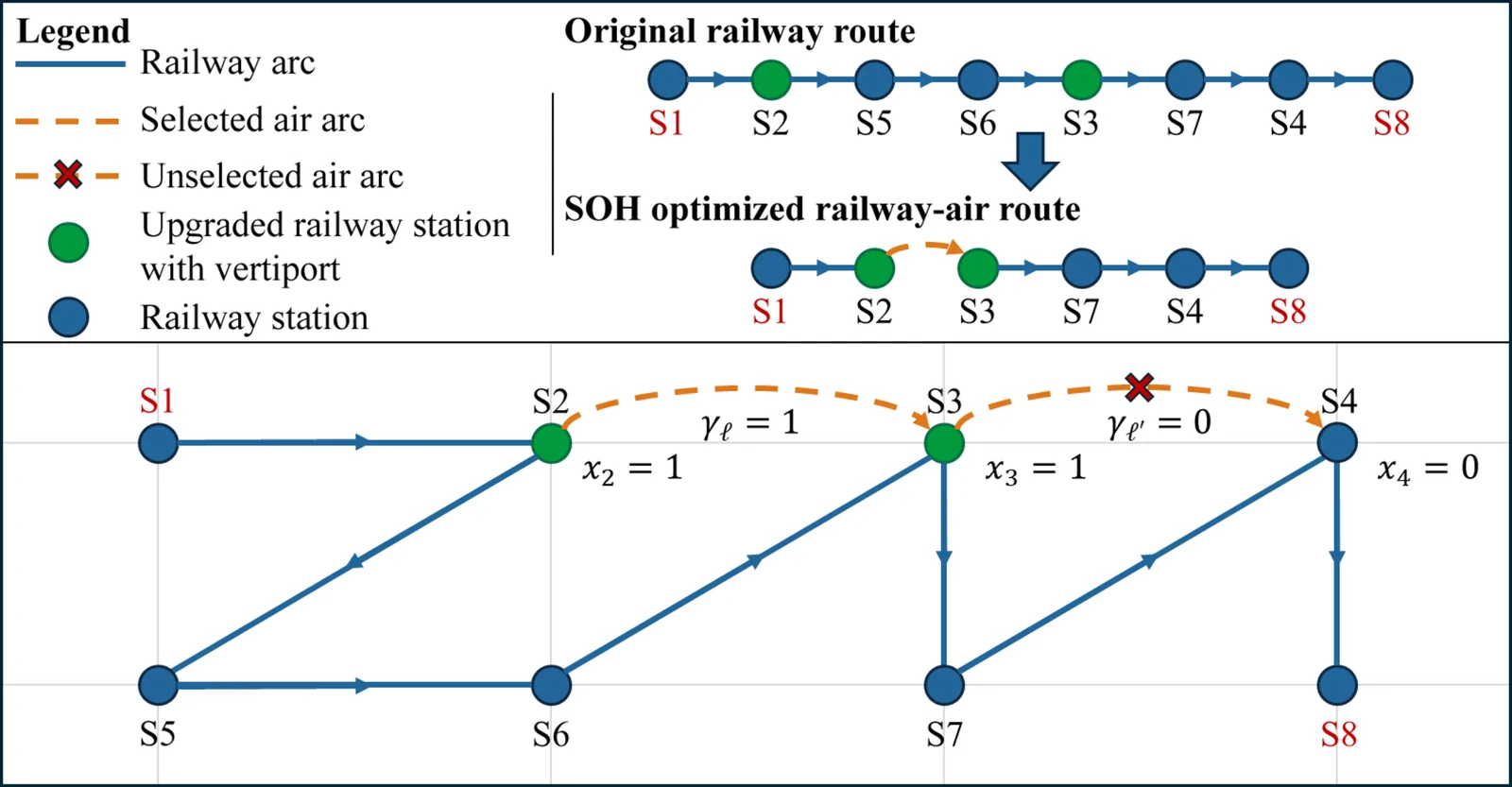
Illustration of the one-hop RAR substitution structure under the SOH formulation. The upper panel compares the original railway route with the SOH optimized route with a one-hop air arc. The lower panel shows the candidate schemes and binary scheme selection, where transferable demand is assigned through selected one-hop substitution schemes between vertiports.

This restriction serves both computational and behavioral purposes. It reduces the route set to candidate schemes and represents a simple travel pattern with rail access, one direct eVTOL segment, and rail egress. By excluding repeated changes between rail and air, the structure is consistent with evidence that passengers tend to avoid routes with numerous transfers [50].

For each OD pair p∈P with origin $o_{p}$ and destination $d_{p}$, the SOH formulation restricts the admissible substitution structure of mobile demand to

$$o_{p}\overset{\text{rail}}{\to }a\overset{\text{air}}{\to }b\overset{\text{rail}}{\to }d_{p}$$ (38)

where $\left(a,\;b\right)\in A^{A}$ is the single air arc used by the trip.

Let $D_{\mathrm{ij}}^{R}$ denote the shortest path generalized railway cost between stations *i* and *j*. Then, for OD pair *p* and feasible air arc $\left(a,\;b\right)$, the generalized cost of the corresponding one-hop substitution scheme is

$$\begin{array}{c} C_{\mathrm{pab}}^{\left(2\right)}=D_{o_{p}a}^{R}+c_{\mathrm{ab}}^{A}+D_{{\mathrm{bd}}_{p}}^{R},\;\left(a,\;b\right)\in A^{A} \end{array}$$ (39)

where $c_{\mathrm{ab}}^{A}$ is defined in Eq. (13).

Relative to the all-rail baseline

$$C_{p}^{R}=D_{o_{p}d_{p}}^{R}$$ (40)

the generalized cost change induced by scheme $\left(a,\;b\right)$ is

$$\Delta C_{\mathrm{pab}}=D_{o_{p}a}^{R}+c_{\mathrm{ab}}^{A}+D_{{\mathrm{bd}}_{p}}^{R}-D_{o_{p}d_{p}}^{R}$$ (41)

A negative value of $\Delta C_{\mathrm{pab}}$ means that the one-hop substitution scheme yields a lower generalized travel cost than the all-rail baseline for the mobile demand component of OD pair *p*.

To keep the formulation compact, only beneficial candidate schemes are retained. Specifically, scheme $\left(p,\;a,\;b\right)$ is included only if

$$\Delta C_{\mathrm{pab}}<0$$ (42)

Let $L_{p}$ denote the retained scheme set for OD pair *p*, and let

$$L=\underset{p\in P}{\cup }L_{p}$$ (43)

denote the overall candidate set. For each candidate scheme ℓ∈L, define the binary selection variable

$$\begin{array}{c} y_{\ell }=\left\{\begin{array}{ll} 1, & \text{if candidate scheme }\ell \text{ is selected,}\\ 0, & \text{otherwise} \end{array}\right. \end{array}$$ (44)

A candidate scheme can be selected only if both endpoint stations of its air arc are developed as vertiports

$$y_{\ell }\leq x_{a_{\ell }},\;\forall \ell \in L$$ (45)

$$y_{\ell }\leq x_{b_{\ell }},\;\forall \ell \in L$$ (46)

Under the common transferable demand approximation introduced in the Generalized travel cost and total cost formulation section, the passenger volume associated with a selected scheme is fixed at

$$q_{\ell }=T_{p\left(\ell \right)}^{\mathrm{mob}}$$ (47)

The transferable demand induced by selected schemes is aggregated on each air arc. Hence, for every $\left(a,\;b\right)\in A^{A}$,

$$\begin{array}{c} \sum_{\ell \in L:\left(a_{\ell \;},b_{\ell }\right)=\left(a,b\right)}T_{p\left(\ell \right)}^{\mathrm{mob}}y_{\ell }\leq Q\;n_{\mathrm{ab}},\;\forall \left(a,\;b\right)\in A^{A} \end{array}$$ (48)

Compared with the MCF formulation, Eq. (48) avoids explicit OD by arc flow variables and instead aggregates the contribution of selected candidate schemes directly at the air arc level. This is the main reason why the SOH formulation is more compact for repeated scenario analysis.

Moreover, each OD pair may activate at most one one-hop substitution scheme

$$\sum_{\ell \in L_{p}}y_{\ell }\leq 1,\;\forall p\in P$$ (49)

The SOH scheme selection constraint ensures that the potentially transferable demand of each OD pair either remains entirely on the railway network or is shifted through a one-hop RAR pattern. Travel, infrastructure, and operating costs jointly determine whether a scheme is selected. The SOH formulation is obtained by combining Eqs. (42) to (49) with the infrastructure and operational feasibility [Eqs. (21) to (30)] in the Infrastructure and operational constraints section. The resulting formulation is also a MILP.

Since the SOH formulation evaluates each retained scheme relative to the all-rail baseline, the mobile demand travel cost is conveniently expressed in incremental form. The all-rail baseline cost associated with the mobile demand component is

$$C^{\mathrm{mob},\text{base}}=\sum_{p\in P}T_{p}^{\mathrm{mob}}D_{o_{p}d_{p}}^{R}$$ (50)

If scheme ℓ is selected, then the associated transferable demand volume $T_{p\left(\ell \right)}^{\mathrm{mob}}$ experiences the cost change $\Delta C_{\ell }$. Therefore, the optimized mobile demand travel cost under the SOH formulation is

$$C_{\left(2\right)}^{\mathrm{mob}}=C^{\mathrm{mob},\text{base}}+\sum_{\ell \in L}T_{p\left(\ell \right)}^{\mathrm{mob}}\Delta C_{\ell }y_{\ell }$$ (51)

Accordingly, the total objective of the SOH formulation becomes

$$Z^{\left(2\right)}=C^{\mathrm{fix}}+C_{\left(2\right)}^{\mathrm{mob}}+C^{\text{infra}}+C^{\mathrm{op}}$$ (52)

As in the MCF formulation, $C^{\mathrm{fix}}$ is constant for a given demand set and is included to keep the total cost evaluation fully consistent with the total objective in the Generalized travel cost and total cost formulation section.

The SOH formulation is a structured and computationally efficient approximation for repeated scenario analysis. It concentrates the admissible travel set on simple RAR travel patterns with one direct air segment, thereby avoiding repeated changes between rail and air and replacing explicit flows for each OD pair on every arc with candidate selection. This structure reduces model size while preserving the planning interactions among passenger demand, vertiport activation, capacity, flight frequency, and fleet scale. MCF provides the detailed formulation when broader routing on the integrated network is required.

## Data Availability

Data will be made available upon reasonable request.
