# Supplementary material for: Railway-Integrated Planning of Electric Vertical Takeoff and Landing Aircraft (eVTOL) Vertiports
Source: Research (Wash D C). 2026 Oct 23;9:1453. doi: 10.34133/research.1453 (PMC13691265; doi:10.34133/research.1453)
Supplement: Supplementary file 1 [file research.1453.f1.pdf]

Supplementary Materials

Table S1. Station dataset used in the Guangdong case study

Population is aggregated from the WorldPop China population raster to the nearest railway station through the Voronoi partition.

Station ID	Station Name	Longitude (WGS84)	Latitude (WGS84)	Aggregated Population
1	Chaoshan Station	116.583575	23.539379	1,690,384
2	Raoping Station	116.928733	23.714379	987,752
3	Humen Station	113.668295	22.864348	2,241,842
4	Dongguan Station	113.853979	23.090335	3,182,650
5	Dongguan East Station	114.033952	22.969893	2,054,035
6	Sanshui South Station	112.866282	23.116972	1,282,185
7	Foshan Station	113.098416	23.049878	2,489,306
8	Shunde College Station	113.314216	22.825507	1,693,532
9	Guangzhou North Station	113.199136	23.379599	1,904,119
10	Guangzhou Station	113.252242	23.151582	1,704,907
11	Guangzhou South Station	113.264122	22.991459	1,724,385
12	Guangzhou East Station	113.319170	23.152693	2,562,616
13	Heyuan Station	114.678540	23.762084	1,302,721
14	Heping Station	114.964084	24.460519	763,325
15	Longchuan Station	115.232621	24.115060	828,747
16	Huizhou Station	114.411621	23.154265	1,728,102
17	Huidong Station	114.789929	22.846909	781,608
18	Enping Station	112.395439	22.185128	687,354
19	Kaiping South Station	112.674890	22.319419	1,281,970
20	Jiangmen Station	113.097804	22.563665	2,380,709
21	Puning Station	116.194832	23.269274	2,979,619
22	Jieyang Station	116.365121	23.572747	1,782,480
23	Huazhou Station	110.647312	21.655872	1,362,860
24	Maoming Station	110.843485	21.683697	3,665,402
25	Xingning Station	115.723950	24.105162	1,678,611
26	Meizhou Station	116.126468	24.261733	1,438,542
27	Qingyuan Station	113.129010	23.696526	1,413,685
28	Yingde West Station	113.345093	24.162602	1,160,123
29	Chaoyang Station	116.417305	23.383736	2,078,930
30	Shantou Station	116.750151	23.374581	1,971,212
31	Shanwei Station	115.423704	22.813301	1,014,633
32	Lufeng Station	115.728464	22.950765	1,525,628
33	Lechang Station	113.351569	25.132015	613,450

Station ID	Station Name	Longitude (WGS84)	Latitude (WGS84)	Aggregated Population
34	Shaoguan East Station	113.599162	24.797119	1,573,307
35	Guangmingcheng Station	113.949612	22.736195	2,743,201
36	Shenzhen North Station	114.023922	22.611958	2,377,016
37	Shenzhen Station	114.112069	22.534299	971,915
38	Shenzhen Pingshan Station	114.321406	22.710511	2,000,526
39	Yangjiang Station	111.990170	21.810380	1,199,775
40	Yangchun Station	111.756315	22.192776	1,406,731
41	Yunan Station	111.464706	23.248108	951,287
42	Yunfu East Station	112.133788	23.014393	1,103,302
43	Leizhou Station	110.041764	20.921502	1,955,082
44	Zhanjiang West Station	110.276244	21.218425	1,901,865
45	Hechun Station	110.304149	21.704887	1,286,115
46	Huaiji Station	112.154215	23.895341	1,635,659
47	Zhaoqing Station	112.442563	23.084038	908,694
48	Zhaoqing East Station	112.658350	23.216214	672,560
49	Zhongshan Station	113.427760	22.546492	1,497,067
50	Zhuhai Station	113.544287	22.218163	1,366,957

Table S2. Railway link connections between station pairs

The link list defines the railway topology used in the Guangdong case study.

Link ID	From Station	To Station
1	Chaoshan Station	Raoping Station
2	Chaoshan Station	Shantou Station
3	Humen Station	Guangzhou South Station
4	Dongguan Station	Guangzhou East Station
5	Dongguan East Station	Guangzhou South Station
6	Dongguan East Station	Dongguan Station
7	Sanshui South Station	Zhaoqing East Station
8	Foshan Station	Guangzhou South Station
9	Foshan Station	Yangchun Station
10	Foshan Station	Sanshui South Station
11	Shunde College Station	Guangzhou South Station
12	Shunde College Station	Zhongshan Station
13	Guangzhou North Station	Guangzhou Station
14	Guangzhou Station	Guangzhou South Station
15	Guangzhou Station	Foshan Station
16	Guangzhou East Station	Guangzhou Station
17	Heyuan Station	Huizhou Station
18	Heping Station	Longchuan Station
19	Longchuan Station	Heyuan Station
20	Huizhou Station	Dongguan East Station
21	Huizhou Station	Guangzhou East Station
22	Huidong Station	Huizhou Station
23	Enping Station	Yangjiang Station
24	Kaiping South Station	Enping Station
25	Jiangmen Station	Kaiping South Station
26	Puning Station	Lufeng Station
27	Jieyang Station	Chaoshan Station
28	Jieyang Station	Meizhou Station
29	Huazhou Station	Maoming Station
30	Maoming Station	Zhanjiang West Station
31	Xingning Station	Longchuan Station
32	Meizhou Station	Xingning Station
33	Qingyuan Station	Guangzhou North Station
34	Yingde West Station	Qingyuan Station
35	Chaoyang Station	Chaoshan Station

Link ID	From Station	To Station
36	Chaoyang Station	Puning Station
37	Shantou Station	Shanwei Station
38	Shanwei Station	Shenzhen Pingshan Station
39	Shanwei Station	Huidong Station
40	Lufeng Station	Shanwei Station
41	Lechang Station	Shaoguan East Station
42	Shaoguan East Station	Yingde West Station
43	Guangmingcheng Station	Humen Station
44	Shenzhen North Station	Guangmingcheng Station
45	Shenzhen Station	Shenzhen North Station
46	Shenzhen Station	Dongguan East Station
47	Shenzhen Pingshan Station	Shenzhen North Station
48	Yangjiang Station	Maoming Station
49	Yangchun Station	Maoming Station
50	Yunan Station	Yunfu East Station
51	Yunfu East Station	Zhaoqing East Station
52	Leizhou Station	Zhanjiang West Station
53	Zhanjiang West Station	Hechun Station
54	Hechun Station	Huazhou Station
55	Huaiji Station	Zhaoqing East Station
56	Zhaoqing Station	Zhaoqing East Station
57	Zhongshan Station	Jiangmen Station
58	Zhuhai Station	Zhongshan Station

Table S3. eVTOL flight and OD demand parameters

The table reports the flight, charging, demand, and sensitivity parameters used in the analyses.

Parameter	Value	Unit / description
Gravity model distance decay exponent alpha	1.3	-
Total daily OD demand T_tot	612,883	pax/day; calibrated scenario demand
Transferable demand ratio pi	0.05 baseline; {0.01, 0.02, 0.05, 0.10, 0.15, 0.20}	Scenario parameter and sensitivity values
Seats per flight Q	4	seats/flight
Minimum feasible eVTOL flight distance R_min	50	km
Maximum feasible eVTOL flight distance R_max	160 baseline; {120, 130, 160, 180, 200}	km; five values in the sensitivity analysis
Usable battery capacity B_use	120 baseline; {80, 100, 120, 160, 200}	kWh; five values in the air arc feasibility analysis
Battery reserve fraction rho_res	0.20	-; reserve fraction for air arc feasibility
Takeoff time t_TO	0.5	min
Climb time t_climb	2	min
Descent time t_desc	2	min
Landing time t_LD	0.5	min
Pad occupation time before departure t_prep	0.12	h
Turnaround time after arrival t_turn	6 baseline; {4, 5, 6, 8, 10}	min; five values in the sensitivity analysis
Cruise speed v_cruise	320	km/h
Cruise power P_cruise	171	kW
Charging efficiency eta_chg	0.92	-
Rated charging power P_chg	300 baseline; {150, 220, 300, 420, 500}	kW; five values in the sensitivity analysis
Takeoff power P_TO	180	kW
Climb power P_climb	150	kW
Descent power P_desc	120	kW
Landing power P_LD	180	kW

Table S4. Optimization parameter settings

The table reports the site limit, cost, capacity, and solver settings used in the analyses. Economic sensitivity tests vary one factor at a time while keeping the remaining inputs fixed.

Parameter	Value	Unit / description
Site budget upper bound Kmax	20 main comparison; {5, 10, 12, 15, 20}	sum_i x_i <= Kmax
Railway operating speed v_R	160	km/h
Value of time VOT	120 baseline; {60, 90, 120, 150, 180}	CNY/h; five values in the sensitivity analysis
Railway circuity factor	1.25	Correction from straight-line distance to railway distance
Railway stop penalty t_stop	7/60	h per traversed railway link
Daily operating window H	12	h/day
Peak demand amplification factor K_peak	1.348	-
Pad capacity upper bound m_bar,i	8	pads/site
Charging capacity upper bound s_bar,i	2,000	kW/site
Maximum pad utilization rho_m	0.85	-
Maximum fleet utilization rho_F	0.85	-
Fixed vertiport investment c_x	5 x 10^6 baseline; {2.5, 3.75, 5, 6.25, 7.5} x 10^6	CNY/site; five values in the sensitivity analysis
Pad investment cost c_m	8 x 10^5 baseline; {4, 6, 8, 10, 12} x 10^5	CNY/pad; five values in the sensitivity analysis
Charging capacity investment cost c_s	2,000 baseline; {1,000, 1,500, 2,000, 2,500, 3,000}	CNY/kW; five values in the sensitivity analysis
Fleet investment cost c_F	2 x 10^6 baseline; {1, 1.5, 2, 2.5, 3} x 10^6	CNY/aircraft; five values in the sensitivity analysis
Flight operating cost c_op,ab	Route dependent	CNY/flight on air arc (a,b)
Annual discount rate	0.08	-
Planning horizon	10	years
Operating days	365	days/year
Fixed site O&M cost c_om	5,000	CNY/site/day
Maximum station throughput $\bar{\mu}$	15	aircraft movements/hour/site
Flight dispatch cost c_disp	80 baseline; {40, 60, 80, 100, 120}	CNY/flight; five values in the sensitivity analysis
Flight time cost c_time	180 baseline; {90, 135, 180, 225, 270}	CNY/(flight h); varied with c_maint
Flight maintenance cost c_maint	220 baseline; {110, 165, 220, 275, 330}	CNY/(flight h); varied with c_time
Battery throughput cost c_bat	0.35 baseline; {0.15, 0.25, 0.35, 0.45, 0.55}	CNY/kWh; five values in the sensitivity analysis
Grid energy cost c_ene	1.5 baseline; {0.7, 1.1, 1.5, 1.9, 2.3}	CNY/kWh drawn from charger; five values in the sensitivity analysis
Flight cost based on distance c_km	1	CNY/(flight km)
Paired sensitivity design for individual factors	13 factors; 5 values/factor; MCF and SOH	106 unique results across the formulations and scenarios; the common baseline is repeated only for display by factor

Table S5. Aircraft and charging sensitivity results

Each factor uses five input values under the 50-node paired sensitivity baseline (Kmax = 20, pi = 0.05, Rmax = 160 km, usable battery = 120 kWh, and reserve = 20%) and is solved with both MCF and SOH. Both formulations use a relative MIP gap target of 0.1%. The gap columns report the final relative MIP gaps as percentages. The common baseline is solved once per formulation and repeated only for display. Battery cases apply the air arc feasibility condition after the reserve is deducted. Benefits are reported in CNY/day, with changes relative to the corresponding formulation baseline. The site Jaccard index compares the sets selected by MCF and SOH at the same input value.

Varied factor	Input value	MCF benefit (change)	MCF sites / fleet	SOH benefit (change)	SOH sites / fleet	Site Jaccard (MCF vs SOH)	MCF gap (%)	SOH gap (%)
Usable battery capacity	80 kWh	34,562 (-15.0%)	9 / 41	36,001 (-12.7%)	7 / 29	0.60	0.072	0.058
Usable battery capacity	100 kWh	37,853 (-6.9%)	13 / 60	39,729 (-3.7%)	12 / 59	0.92	0.100	0.089
Usable battery capacity	120 kWh	40,674 (+0.0%)	13 / 61	41,256 (+0.0%)	12 / 59	0.92	0.080	0.099
Usable battery capacity	160 kWh	40,674 (+0.0%)	13 / 61	41,256 (+0.0%)	12 / 59	0.92	0.080	0.099
Usable battery capacity	200 kWh	40,674 (+0.0%)	13 / 61	41,256 (+0.0%)	12 / 59	0.92	0.080	0.099
Rated charging power	150 kW	27,574 (-32.2%)	8 / 44	28,782 (-30.2%)	8 / 43	1.00	0.051	0.051
Rated charging power	220 kW	34,202 (-15.9%)	13 / 64	35,553 (-13.8%)	12 / 63	0.92	0.078	0.067
Rated charging power	300 kW	40,674 (+0.0%)	13 / 61	41,256 (+0.0%)	12 / 59	0.92	0.080	0.099
Rated charging power	420 kW	44,920 (+10.4%)	13 / 57	47,348 (+14.8%)	12 / 57	0.92	0.100	0.067
Rated charging power	500 kW	47,533 (+16.9%)	13 / 55	49,450 (+19.9%)	12 / 57	0.92	0.100	0.099
Operational range	120 km	33,273 (-18.2%)	12 / 50	36,001 (-12.7%)	7 / 29	0.58	0.095	0.092
Operational range	130 km	35,047 (-13.8%)	12 / 51	36,600 (-11.3%)	9 / 43	0.75	0.100	0.012
Operational range	160 km	40,674 (+0.0%)	13 / 61	41,256 (+0.0%)	12 / 59	0.92	0.080	0.099
Operational range	180 km	40,674 (+0.0%)	13 / 61	42,571 (+3.2%)	11 / 60	0.85	0.100	0.097
Operational range	200 km	40,674 (+0.0%)	13 / 61	42,571 (+3.2%)	11 / 60	0.85	0.100	0.097
Turnaround time after arrival	4 min	42,891 (+5.5%)	14 / 63	44,909 (+8.9%)	13 / 63	0.80	0.099	0.066
Turnaround time after arrival	5 min	41,490 (+2.0%)	13 / 60	42,838 (+3.8%)	12 / 58	0.92	0.100	0.030
Turnaround time after arrival	6 min	40,674 (+0.0%)	13 / 61	41,256 (+0.0%)	12 / 59	0.92	0.080	0.099
Turnaround time after arrival	8 min	36,568 (-10.1%)	12 / 58	38,389 (-6.9%)	11 / 58	0.92	0.091	0.099
Turnaround time after arrival	10 min	34,108 (-16.1%)	10 / 54	35,401 (-14.2%)	12 / 64	0.83	0.086	0.095

Table S6. Economic sensitivity results

Each economic factor uses five input values under the 50-node paired sensitivity baseline (Kmax = 20, pi = 0.05, Rmax = 160 km, usable battery = 120 kWh, and reserve = 20%) and is solved with both MCF and SOH. Both formulations use a relative MIP gap target of 0.1%. The gap columns report the final relative MIP gaps as percentages. The common baseline is solved once per formulation and repeated only for display. The time and maintenance case scales both hourly coefficients together. Benefits are reported in CNY/day, with changes relative to the corresponding formulation baseline. The site Jaccard index compares the sets selected by MCF and SOH at the same input value.

Varied factor	Input value	MCF benefit (change)	MCF sites / fleet	SOH benefit (change)	SOH sites / fleet	Site Jaccard (MCF vs SOH)	MCF gap (%)	SOH gap (%)
Fleet investment cost	1 million CNY/aircraft	71,846 (+76.6%)	16 / 87	72,268 (+75.2%)	16 / 87	1.00	0.046	0.094
Fleet investment cost	1.50 million CNY/aircraft	53,127 (+30.6%)	13 / 61	55,279 (+34.0%)	14 / 69	0.93	0.100	0.087
Fleet investment cost	2 million CNY/aircraft	40,674 (+0.0%)	13 / 61	41,256 (+0.0%)	12 / 59	0.92	0.080	0.099
Fleet investment cost	2.50 million CNY/aircraft	28,781 (-29.2%)	7 / 33	31,463 (-23.7%)	8 / 35	0.67	0.098	0.085
Fleet investment cost	3 million CNY/aircraft	22,044 (-45.8%)	7 / 33	24,366 (-40.9%)	8 / 34	0.67	0.085	0.088
Battery throughput cost	0.15 CNY/kWh	47,212 (+16.1%)	13 / 61	48,736 (+18.1%)	14 / 69	0.93	0.100	0.080
Battery throughput cost	0.25 CNY/kWh	43,943 (+8.0%)	13 / 61	44,965 (+9.0%)	14 / 69	0.93	0.100	0.058
Battery throughput cost	0.35 CNY/kWh	40,674 (+0.0%)	13 / 61	41,256 (+0.0%)	12 / 59	0.92	0.080	0.099
Battery throughput cost	0.45 CNY/kWh	37,405 (-8.0%)	13 / 61	38,112 (-7.6%)	12 / 59	0.92	0.100	0.085
Battery throughput cost	0.55 CNY/kWh	34,136 (-16.1%)	13 / 61	35,235 (-14.6%)	10 / 49	0.77	0.100	0.091
Charging capacity investment cost	1,000 CNY/kW	42,173 (+3.7%)	13 / 61	42,923 (+4.0%)	14 / 69	0.93	0.100	0.080
Charging capacity investment cost	1,500 CNY/kW	41,423 (+1.8%)	13 / 61	42,059 (+1.9%)	14 / 69	0.93	0.100	0.062
Charging capacity investment cost	2,000 CNY/kW	40,674 (+0.0%)	13 / 61	41,256 (+0.0%)	12 / 59	0.92	0.080	0.099
Charging capacity investment cost	2,500 CNY/kW	39,924 (-1.8%)	13 / 61	40,535 (-1.7%)	12 / 59	0.92	0.100	0.099
Charging capacity investment cost	3,000 CNY/kW	39,175 (-3.7%)	13 / 61	39,810 (-3.5%)	12 / 59	0.92	0.100	0.082
Flight dispatch cost	40 CNY/flight	71,435 (+75.6%)	16 / 84	73,356 (+77.8%)	16 / 82	1.00	0.083	0.095
Flight dispatch cost	60 CNY/flight	54,724 (+34.5%)	16 / 83	55,999 (+35.7%)	15 / 78	0.94	0.092	0.093
Flight dispatch cost	80 CNY/flight	40,674 (+0.0%)	13 / 61	41,256 (+0.0%)	12 / 59	0.92	0.080	0.099
Flight dispatch cost	100 CNY/flight	28,655 (-29.5%)	11 / 55	30,362 (-26.4%)	8 / 41	0.73	0.089	0.033
Flight dispatch cost	120 CNY/flight	21,320 (-47.6%)	8 / 38	22,407 (-45.7%)	7 / 32	0.67	0.051	0.013
Grid energy cost	0.70 CNY/kWh	75,669 (+86.0%)	20 / 103	78,373 (+90.0%)	18 / 96	0.90	0.100	0.099
Grid energy cost	1.10 CNY/kWh	54,886 (+34.9%)	13 / 61	57,631 (+39.7%)	15 / 78	0.87	0.100	0.095
Grid energy cost	1.50 CNY/kWh	40,674 (+0.0%)	13 / 61	41,256 (+0.0%)	12 / 59	0.92	0.080	0.099
Grid energy cost	1.90 CNY/kWh	29,029 (-28.6%)	10 / 49	30,901 (-25.1%)	8 / 35	0.64	0.069	0.084
Grid energy cost	2.30 CNY/kWh	21,456 (-47.2%)	8 / 32	23,725 (-42.5%)	7 / 29	0.88	0.058	0.085
Time and maintenance cost multiplier	0.50 times baseline	96,597 (+137.5%)	20 / 114	98,535 (+138.8%)	19 / 110	0.95	0.086	0.079

Varied factor	Input value	MCF benefit (change)	MCF sites / fleet	SOH benefit (change)	SOH sites / fleet	Site Jaccard (MCF vs SOH)	MCF gap (%)	SOH gap (%)
Time and maintenance cost multiplier	0.75 times baseline	62,678 (+54.1%)	16 / 82	65,573 (+58.9%)	16 / 86	0.88	0.099	0.067
Time and maintenance cost multiplier	1 times baseline	40,674 (+0.0%)	13 / 61	41,256 (+0.0%)	12 / 59	0.92	0.080	0.099
Time and maintenance cost multiplier	1.25 times baseline	27,556 (-32.3%)	8 / 35	27,706 (-32.8%)	8 / 35	1.00	0.012	0.080
Time and maintenance cost multiplier	1.50 times baseline	14,960 (-63.2%)	5 / 18	18,943 (-54.1%)	2 / 10	0.40	0.100	0.053
Pad investment cost	0.40 million CNY/pad	47,043 (+15.7%)	13 / 61	48,041 (+16.4%)	14 / 69	0.93	0.100	0.079
Pad investment cost	0.60 million CNY/pad	43,859 (+7.8%)	13 / 61	44,621 (+8.2%)	14 / 69	0.93	0.100	0.083
Pad investment cost	0.80 million CNY/pad	40,674 (+0.0%)	13 / 61	41,256 (+0.0%)	12 / 59	0.92	0.080	0.099
Pad investment cost	1 million CNY/pad	37,489 (-7.8%)	13 / 61	38,216 (-7.4%)	12 / 59	0.92	0.100	0.099
Pad investment cost	1.20 million CNY/pad	34,304 (-15.7%)	13 / 61	35,418 (-14.2%)	10 / 49	0.77	0.100	0.036
Fixed vertiport investment	2.50 million CNY/site	53,944 (+32.6%)	13 / 61	55,474 (+34.5%)	14 / 69	0.93	0.100	0.100
Fixed vertiport investment	3.75 million CNY/site	47,309 (+16.3%)	13 / 61	48,339 (+17.2%)	14 / 69	0.93	0.100	0.054
Fixed vertiport investment	5 million CNY/site	40,674 (+0.0%)	13 / 61	41,256 (+0.0%)	12 / 59	0.92	0.080	0.099
Fixed vertiport investment	6.25 million CNY/site	34,039 (-16.3%)	13 / 61	35,447 (-14.1%)	11 / 55	0.85	0.100	0.081
Fixed vertiport investment	7.50 million CNY/site	29,832 (-26.7%)	9 / 46	31,010 (-24.8%)	8 / 41	0.70	0.091	0.057
Value of time	60 CNY/h	0 (-100.0%)	0 / 0	0 (-100.0%)	0 / 0	1.00	0.044	0.000
Value of time	90 CNY/h	1,720 (-95.8%)	2 / 11	2,653 (-93.6%)	2 / 9	1.00	0.076	0.000
Value of time	120 CNY/h	40,674 (+0.0%)	13 / 61	41,256 (+0.0%)	12 / 59	0.92	0.080	0.099
Value of time	150 CNY/h	214,906 (+428.4%)	20 / 124	216,359 (+424.4%)	20 / 128	1.00	0.079	0.085
Value of time	180 CNY/h	427,610 (+951.3%)	20 / 133	426,179 (+933.0%)	20 / 131	1.00	0.085	0.096
